# Reactive metabolites of acetaminophen activate and sensitize the capsaicin receptor TRPV1

**DOI:** 10.1038/s41598-017-13054-3

**Published:** 2017-10-06

**Authors:** Mirjam J. Eberhardt, Florian Schillers, Esther M. Eberhardt, Linus Risser, Jeanne de la Roche, Christine Herzog, Frank Echtermeyer, Andreas Leffler

**Affiliations:** 1 Department of Anaesthesiology and Intensive Care Medicine, Hannover Medical School, Hannover, Carl-Neuberg-Strasse 1, 30625 Hannover, Germany; 2 Department of Anaesthesiology, Friedrich-Alexander-University Erlangen-Nuremberg, Krankenhausstrasse 12, 91054 Erlangen, Germany; 3 Institute for Neurophysiology, Hannover Medical School, Carl-Neuberg-Strasse 1, 30625 Hannover, Germany

## Abstract

The irritant receptor TRPA1 was suggested to mediate analgesic, antipyretic but also pro-inflammatory effects of the non-opioid analgesic acetaminophen, presumably due to channel activation by the reactive metabolites parabenzoquinone (pBQ) and N-acetyl-parabenzoquinonimine (NAPQI). Here we explored the effects of these metabolites on the capsaicin receptor TRPV1, another redox-sensitive ion channel expressed in sensory neurons. Both pBQ and NAPQI, but not acetaminophen irreversibly activated and sensitized recombinant human and rodent TRPV1 channels expressed in HEK 293 cells. The reducing agents dithiothreitol and N-acetylcysteine abolished these effects when co-applied with the metabolites, and both pBQ and NAPQI failed to gate TRPV1 following substitution of the intracellular cysteines 158, 391 and 767. NAPQI evoked a TRPV1-dependent increase in intracellular calcium and a potentiation of heat-evoked currents in mouse spinal sensory neurons. Although TRPV1 is expressed in mouse hepatocytes, inhibition of TRPV1 did not alleviate acetaminophen-induced hepatotoxicity. Finally, intracutaneously applied NAPQI evoked burning pain and neurogenic inflammation in human volunteers. Our data demonstrate that pBQ and NAQPI activate and sensitize TRPV1 by interacting with intracellular cysteines. While TRPV1 does not seem to mediate acetaminophen-induced hepatotoxicity, our data identify TRPV1 as a target of acetaminophen with a potential relevance for acetaminophen-induced analgesia, antipyresia and inflammation.

## Introduction

Acetaminophen is one of the most commonly used analgesics worldwide, and it is generally assumed that it induces analgesia and antipyresia mainly by inhibiting cyclooxygenase^1^. However, the exact mode of action is still not fully understood and this also applies to adverse effects of acetaminophen such as hepatotoxicity^2^. Recent studies propose spinal and supraspinal mechanisms of action for acetaminophen, and the transient receptor potential (TRP) channel TRPA1 was suggested to be responsible for the desired analgesic and hypothermic effects^3–5^, but also for pro-inflammatory effects in the airways^6^. TRPA1 is a polymodal non-selective cation channel which can be activated by several irritant substances as well as by both cold and heat^7^. Reactive agonists activate TRPA1 by covalent modification or oxidation of N-terminal cysteines^8,9^, an effect which can be prevented by antioxidants also known to be effective for treatment of acetaminophen-induced liver damage^10,11^. While acetaminophen itself seems to be fairly inert on sensory neurons, the reactive metabolites NAPQI and pBQ activate TRPA1. Furthermore, activation of TRPA1 by at least pBQ also seems to involve a modification of several cysteines^12^. Both metabolites result from a cytochrome P450-dependent metabolism of acetaminophen mainly in the liver, but they can also be detected in the upper laminae of the spinal cord as well as in the lung following systemic administration of acetaminophen^5^. The analgesic efficacy of acetaminophen is strongly reduced in mice lacking TRPA1, and it was suggested that activation of TRPA1 by NAPQI on central nerve terminals results in a pre-synaptic inhibition. While intrathecal injection of acetaminophen, pBQ and NAPQI indeed produces analgesic effects in rodents, intraplantar injections of acetaminophen are ineffective^5^. This finding might be due to the short half-life of NAPQI^13^, requiring a rapid transport to its site of action. However, toxic effects of acetaminophen in tissues like the lung and the kidney have been shown to be at least partly caused by liver-derived acetaminophen metabolites^14^. Accordingly, acetaminophen-induced airway inflammation is diminished in mice lacking TRPA1^15^.

Although not as evident as it is known for TRPA1, the capsaicin and heat receptor TRPV1 can also be sensitized and activated by several reactive substances. It was recently demonstrated that the TRPA1-agonist allylisothiocyanate also gates TRPV1, and oxidants like H_2_O_2_ activate TRPV1^16,17^. Similar to TRPA1, oxidation seems to gate TRPV1 by interacting with specific intracellular cysteines^17^. Besides sharing several functional and pharmacological properties, TRPA1 and TRPV1 are co-expressed in a large population of nociceptive sensory neurons, including sensory nerve endings in the spinal cord^18,19^. Furthermore, expression of TRPV1 in hepatocytes seems to regulate cell viability and thus organ function^20,21^. Based on these considerations, we hypothesized that NAPQI and pBQ may functionally modify TRPV1 as well. In the present study we tested this hypothesis by performing *in vitro* electrophysiology and calcium imaging on recombinant as well as native human or rodent TRPV1 channels. Furthermore, mutagenesis was used to identify the molecular mechanisms that account for the observed effects of both metabolites on TRPV1. Our data clearly indicate that TRPV1 is a relevant target of NAPQI and pBQ.

## Results

### pBQ and NAPQI, but not acetaminophen gate TRPV1

**Figure 1:**
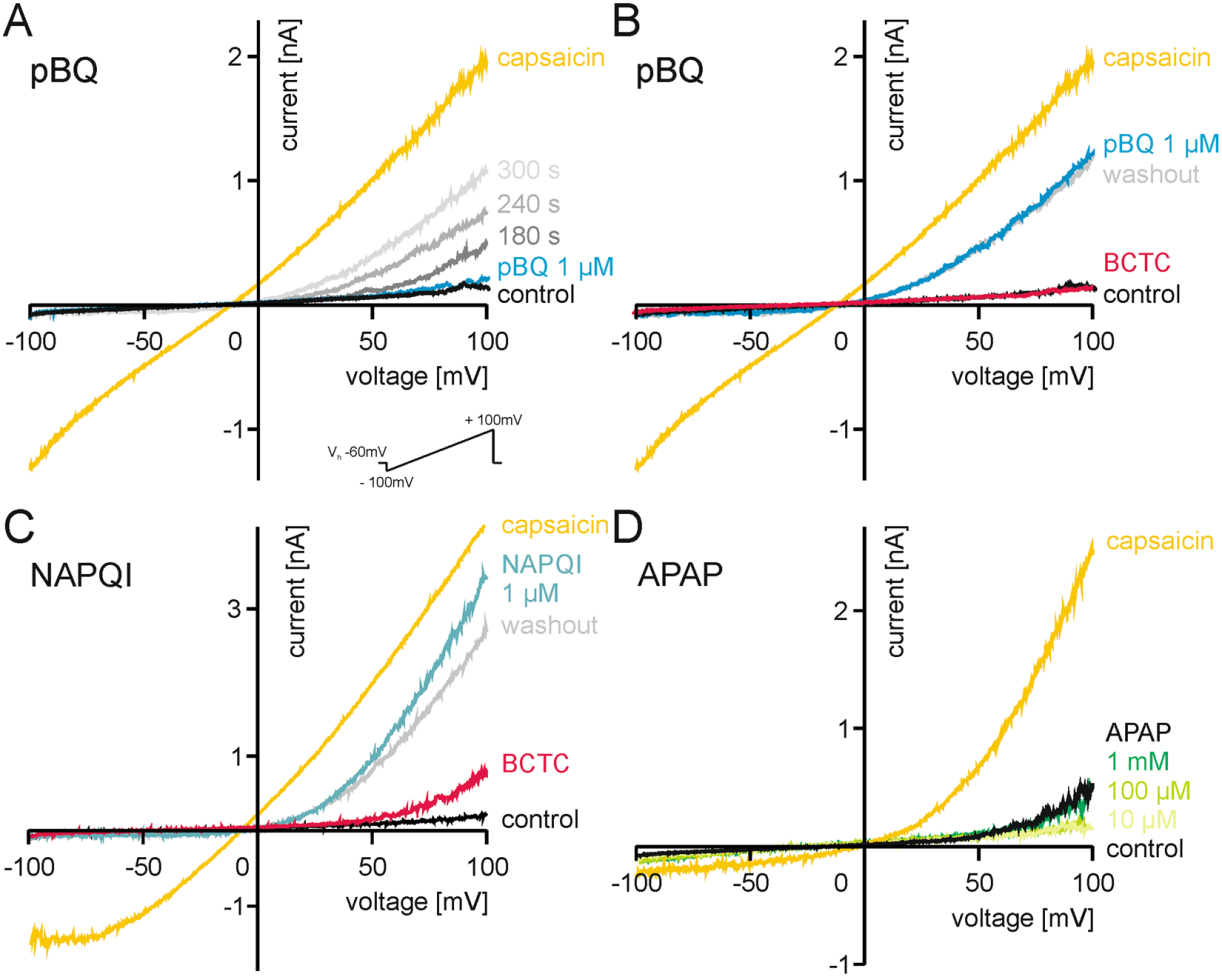
Ramp currents generated by TRPV1 are sensitized by pBQ and NAPQI. Traces show voltage ramp-induced membrane currents (see insert) in hTRPV1-expressing HEK 293 cells, one representative recording out of 6–7 measured cells of each group. (**A**) Sensitization of hTRPV1 by 1 µM pBQ is dependent on the duration of application, a maximum is reached at about 300 s. 1 µM capsaicin was used as control. (**B**) 100 nM BCTC blocks pBQ (5 min)-induced currents. Note that currents slowly recover following washout of BCTC for 2 minutes. (**C**) 1 µM NAPQI (5 min) sensitizes ramp currents generated by TRPV1, and these can be transiently blocked by BCTC. (**D**) Increasing concentrations of acetaminophen (APAP 10 µM, 100 µM, 1000 µM, each applied for 4 minutes) has no effects on ramp currents evoked in hTRPV1-expressing cells.

**Figure 2:**
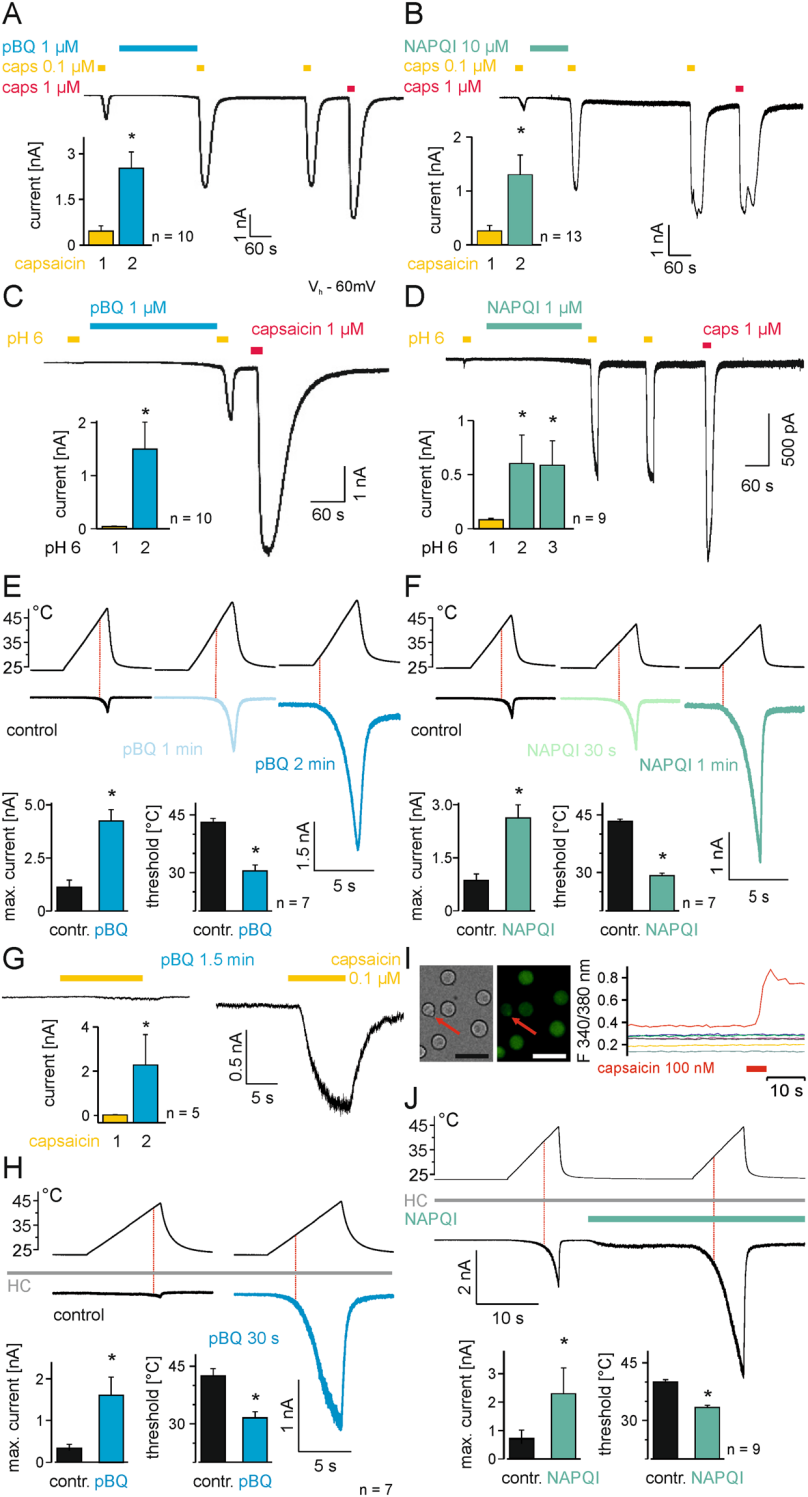
pBQ and NAPQI sensitize TRPV1.Inward currents evoked by 100 nM capsaicin are constantly increased after application of 1 µM pBQ (**A**) or 10 µM NAPQI (**B**) for about 240 s even without their further presence. Proton-induced responses are also sensitized by application of pBQ (**C**) and NAPQI (D). Bar diagrams show means ( ± SEM) of capsaicin- (**A**,**B**) or proton-induced (**C**,**D**) inward currents (*p ≤ 0.02). pBQ (**E**) and NAPQI (**F**) shift the temperature threshold of currents induced by heat ramps (25–45 °C) to lower temperatures, shown as red dotted lines in representative recordings and as bar diagrams of temperature needed to evoke inward currents before and after treatment with pBQ (**E**) and NAPQI (**F**). The maximum heat-induced inward currents are also increased by both metabolites (pBQ *p = 0.028; NAPQI *p = 0.018). Similarly, in DRG neurons of mice pBQ sensitized capsaicin (100 nM) -induced (**G**) and heat induced-currents (**H**) and shifted temperature thresholds to lower temperatures. 50 µM HC 030031 was used throughout these experiments to block TRPA1 (*p = 0.043 (**G**); *p = 0.018 (**H**)). (**I**) A 100 nM capsaicin stimulus was used to identify DRG neurons expressing TRPV1. From left to right: bright field image of small DRG neurons; same neurons loaded with FURA-2AM excited at 340 nm (scale bar = 50 µm); traces of cells during a short application of 100 nM capsaicin. The capsaicin-responding neuron is marked in red and labeled with an arrow. (**J**) Heat-induced currents in TRPV1-expressing neurons are immediately sensitized by 10 µM NAPQI, note that NAPQI itself evokes a small inward current, while TRPA1 was blocked by HC 030031. NAPQI increased heat induced inward currents and shifted temperature thresholds of TRPV1 to lower temperatures (*p = 0.018).

To investigate whether pBQ and NAPQI gate TRPV1, HEK 293 T cells expressing hTRPV1 were examined by whole-cell voltage clamp recordings. Measuring ramp currents by depolarization from −100 to +100 mV applied within 500 ms, 1 µM pBQ activated slowly developing membrane currents with a strong outward rectification in all cells which also generated capsaicin (1 µM)-induced currents due to expression of hTRPV1 (n = 8, Fig. 1A). These currents persisted after washout of pBQ, and could be transiently blocked by the TRPV1-antagonist BCTC (100 nM), i.e. the currents slowly recovered following washout of BCTC (n = 8, Fig. 1B). As demonstrated for pBQ, also 1 µM NAPQI induced slowly developing membrane currents in cells expressing hTRPV1. Again, this effect was inhibited by BCTC (n = 6, Fig. 1C). These persisting currents after wash out of both pBQ and NAPQI indicate an irreversible sensitization of TRPV1. While even low concentrations of pBQ and NAPQI evoked large ramp currents in TRPV1, acetaminophen itself failed to activate membrane currents even in concentrations up to 1 mM applied for several minutes (n = 7, Fig. 1D). A leftward shift of current-voltage curves induced by capsaicin was observed in many experiments following application of pBQ and NAPQI, an effect which can be seen when comparing capsaicin-evoked responses in Fig. 1A–C with Fig. 1D. This effect may indicate that modification of TRPV1 by pBQ and NAPQI is associated with an altered ion permeability, and indeed NAPQI shifted the reversal potential of sodium by about 9 mV (Fig. S1A,B). When constantly holding hTRPV1-expressing cells at −60 mV, 1 µM pBQ evoked small inward currents (62 ± 11 pA (mean ± SEM), n = 29). However, this effect required application of the reactive metabolite for longer than 2 min. (Fig. 2A and C). 3 µM pBQ induced more robust inward currents (389 ± 47 pA) emerging considerably faster. Again, these pBQ-induced membrane currents could be completely but only transiently blocked by BCTC (n = 6, Fig. S2A).

### pBQ and NAPQI sensitize TRPV1 to common agonists

Desensitization of currents evoked by subsequent TRPV1-agonistic stimuli commonly occurs even in calcium free external solution^22^ (Fig. S2B). When a 0.1 µM capsaicin stimulus was followed by a ~4 min long application of 1 µM pBQ however, the induced inward current by a subsequent capsaicin stimulus increased 5.5-fold (p = 0.005, n = 10, Fig. 2A). Notably, this pBQ-evoked sensitization remained stable even after washout of pBQ for several minutes (Fig. 2A). Similarly application of 10 µM NAPQI (~2 min) sensitized capsaicin 0.1µM-activated currents and led to their 11.0-fold increase (p = 0.001; n = 13, Fig. 2B). While application of pH 6.0 for 20 s evoked small inward currents (41 ± 13 pA, n = 8) in cells generating capsaicin (1 µM)-induced membrane currents, the application of 1 µM pBQ resulted in a significant potentiation of pH 6.0-induced inward currents to 1608 ± 559 pA (p = 0.005; Fig. 2C). A similar effect was observed for 1 µM NAPQI, i.e. inward currents induced by pH 6.0 increased from 82 ± 14 pA to 603 ± 264 pA (p = 0.008; n = 9). After wash out of NAPQI for several minutes, proton-evoked currents remained strongly potentiated as compared to the initially evoked current (p = 0.008, Fig. 2D).

Sensitization of TRPV1-mediated heat responses by pBQ and NAPQI was tested by repetitively applying 5 s lasting heat ramps from 25 °C to 45 °C. Indeed, both pBQ and NAPQI strongly enhanced the heat-sensitivity of TRPV1 by shifting the threshold for activation to lower temperatures. The temperature threshold of TRPV1 dropped from 43 ± 1 °C to 31 ± 2 °C by 1 µM pBQ, while heat-induced currents increased from 1124 ± 335 pA to 4247 ± 535 pA (n = 6, p = 0.028 each, Fig. 2E and Fig. S3A). 1 µM NAPQI shifted heat thresholds of hTRPV1 from 43 ± 1 °C to 29 ± 1 °C and enhanced heat-induced inward currents from 860 ± 182 pA to 2630 ± 366 pA (n = 7, p = 0.018 each, Fig. 2F and Fig. S3B, all Wilcoxon matched pairs tests, following Bonferroni correction for comparison of more than 2 groups, p levels considered significant are marked with *).

We now aimed to confirm these sensitizing effects of NAPQI and pBQ on TRPV1 in experiments on mouse DRG neurons. TRPA1, which often co-expressed with TRPV1 in DRG neurons was blocked by 50 µM HC030031. Indeed, 10 µM pBQ sensitized inward currents induced by 100 nM capsaicin within 1.5 minutes from 23 ± 6 pA to 2281 ± 1374 pA (n = 5, p = 0.043, Fig. 2G). Furthermore, in these capsaicin-responsive neurons the temperature threshold of heat-induced inward currents dropped from 42 ± 2 °C to 32 ± 0 °C after a 30 s long application of pBQ. The corresponding inward currents increased from 337 ± 88 pA to 1604 ± 438 pA (n = 7, p = 0.018 each, Fig. 2H). Due to poor stability of NAPQI in water, TRPV1-expressing neurons had to be rapidly identified by using 100 nM capsaicin stimuli in calcium imaging (Fig. 2I). 10 µM NAPQI also induced an immediate sensitization of heat-induced inward currents from 708 ± 305 pA to 2294 ± 906 pA. The temperature thresholds of heat induced currents dropped from 40 ± 1 °C to 33 ± 1 °C (Fig. 2J, n = 7, p = 0.01 each, both Wilcoxon matched pairs test). Neither pBQ nor NAPQI had an effect on heat-evoked effects in capsaicin-insensitive (e.g. TRPV1-neagtive) DRG neurons (S Fig. 4).

### Mechanisms of TRPV1 activation and sensitization by pBQ and NAPQI

**Figure 3:**
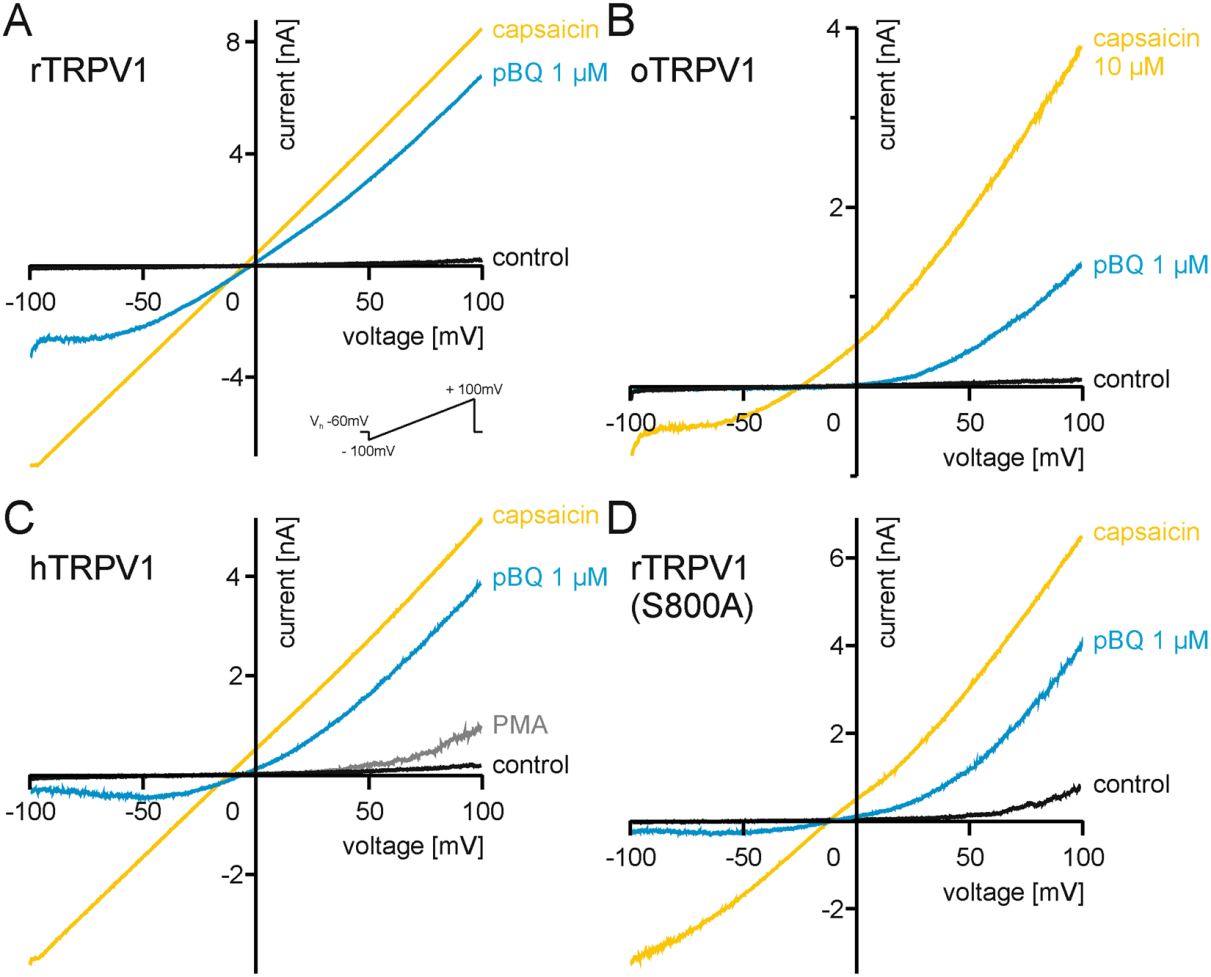
Activation of TRPV1 by pBQ does not involve the capsaicin-binding domain or proteinkinase C-dependent phosphorylation. pBQ (1 µM, 1.5–2 min) sensitized voltage ramp-induced currents in rTRPV1 (**A**) and oTRPV1 (**B**), the latter suggesting a mechanism independent of the capsaicin-binding site. (**C**) Sensitization of hTRPV1 by pBQ (1 µM, 2 min) is additive to sensitization by PKC-dependent phosphorylation evoked by PMA (300 nM). (**D**) In an rTRPV1 mutant lacking the PKC-phosphorylation site S800, membrane currents are still clearly sensitized by pBQ (1 µM 2–3 min).

Similar to the effects on human TRPV1, 1 µM pBQ also sensitized ramp currents in rat TRPV1 (rTRPV1) excluding a species specific mechanism (Fig. 3A, n = 5). Rabbit (o) TRPV1 also shares significant homology with human and rat TRPV1, but displays a 100-fold less sensitivity to vanilloids, thus providing a valuable tool to investigate mechanisms at the capsaicin binding site^23^. As the same low concentration of pBQ (1 µM) sensitized oTRPV1 as well (Fig. 3B, n = 6), a significant effect of pBQ at the vanilloid binding site seems unlikely. Phosphorylation of TRPV1 by protein kinase C (PKC) is a potent mechanism to sensitize or even directly gate TRPV1^24^. Notably, pBQ was described to directly activate PKC^25^. To examine if pBQ interacts with phosphorylation of TRPV1, we next applied the PKC activator PMA (1 µM) for about 4 min in order to completely sensitize the channel by this mechanism. While PMA evoked large membrane currents, the concomitant application of pBQ induced a further potentiation of outwardly rectifying currents (n = 9, Fig. 3C). We confirmed these findings by investigating the mutant construct rTRPV1-S800A at which the phosphorylation site of PKC (Ser 800) was replaced with alanine^24^. On this mutant, 1 µM pBQ still induced large membrane currents (n = 7, Fig. 3D).

**Figure 4:**
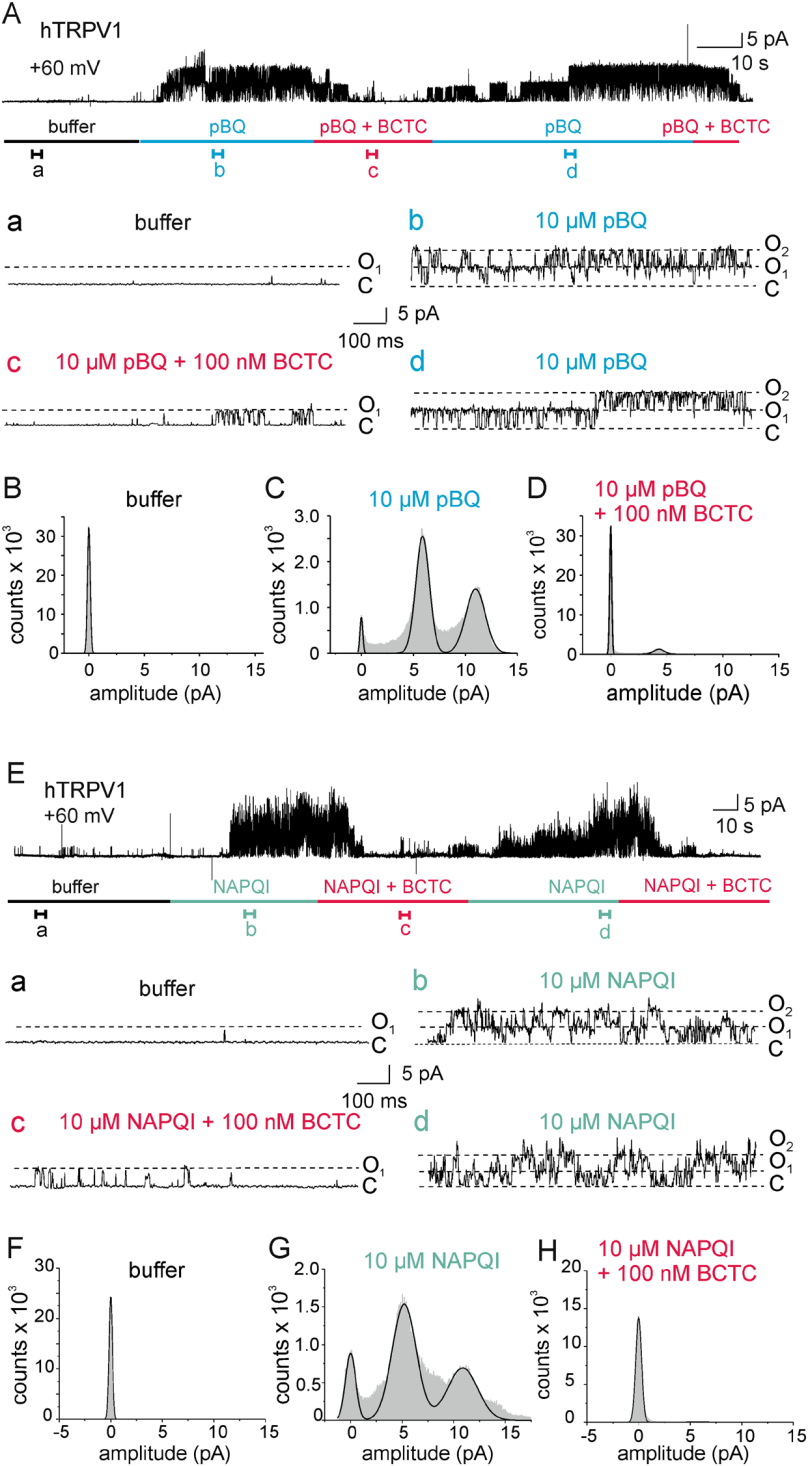
pBQ and NAPQI directly activate TRPV1 from the intracellular side. (**A**) Single channel recordings from inside-out patches show an increased open state probability of TRPV1 channels during 10 µM pBQ application (b) which is decreased by co-application of 100 nM BCTC (c). Sections a,b,c and d represent episodes of 1 s of the upper trace with an expanded time scale. (**B**,**C**,**D**) Amplitude histograms calculated from 20 s sections of the full trace in Fig. 4A show two peaks for the open states of two channels in the patch and a small closed state peak during application of pBQ (**C**). Histograms (**B** and **D**) display a prominent closed state probability of TRPV1 channels before pBQ treatment (buffer, **B**) and after pBQ treatment with co-application of BCTC (**D**). (**E**) Representative trace for single channel recordings from inside-out patches with increased open state probability of TRPV1 during application of 10 µM NAPQI (**b**) and decreased open state probability by co-application of 100 nM BCTC (**c**) Sections a, (b,c) and d represent 1 s episodes of the upper trace with an expanded time scale. (**F**,**G**) and (**H**). Amplitude histograms calculated from 20 s sections of the full trace in Fig. 4E showing two peaks for the open states of two channels in the patch and a small closed state peak during application of NAPQI (**G**). TRPV1 channels reside predominantly in the closed state before NAPQI treatment (buffer, **F**) and during co-application of NAPQI and BCTC (**H**).

**Figure 5:**
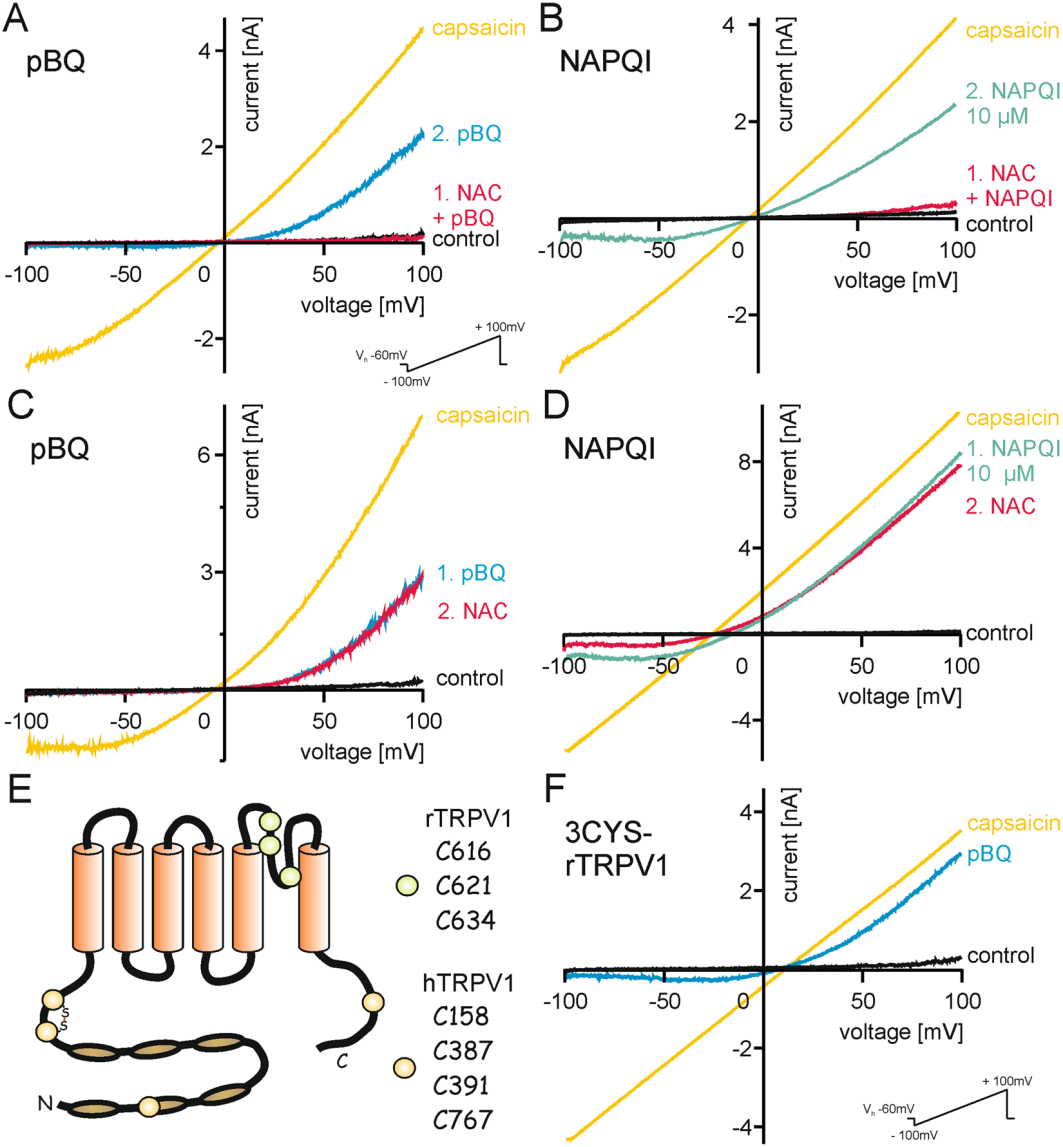
Modulation of cysteines underlies the sensitization of TRPV1 by pBQ and NAPQI. Co-application of 1 mM NAC (5 min) prevents sensitization of ramp currents in hTRPV1 by pBQ (**A**) and NAPQI (**B**). Note that a second application of pBQ (1 µM, 3 min) or NAPQI (10 µM, 1.5 min) alone again increases ramp currents. When NAC (1 mM, 4 min) is applied after sensitization of currents has already been evoked, it fails to reverse the effects of pBQ (**C**) and NAPQI (**D**). (**E**) Schematic model of TRPV1: external cysteines important for sensitization of rTRPV1 by reducing and oxidizing agents^26^ close to the pore forming region are marked with green dots (3CYS-rTRPV1). Internal cysteines of hTRPV1 which have been described to be modulated by oxidative challenges leading to channel sensitization^17^ are marked in yellow. (**F**) pBQ strongly sensitizes the rTRPV1 mutant lacking external cysteines.

In order to determine whether or not pBQ and NAPQI activate TRPV1 channels by mechanisms independent of cytosolic signaling pathways, we next carried out excised inside-out patch clamp recordings. In inside-out macropatches containing TRPV1, both pBQ and NAPQI evoked large membrane currents (S Fig. 5A and B respectively, n = 5 each). In addition, pBQ did not evoke any effects in inside-out patches of untransfected cells (S Fig. 5C, n = 4). Single channel events performed in the inside-out configuration (Fig. 4A,E, n = 5) revealed a high probability for TRPV1 to reside in the open state after application of 10 µM pBQ (Fig. 4A, section b) and 10 µM NAPQI (Fig. 4E, section b). The open state probability was strongly diminished by co-application of 100 nM BCTC together with pBQ (Fig. 4A, section c) or NAPQI (Fig. 4E, section c). After removal of BCTC and continued pBQ/NAPQI application, TRPV1 channels returned to their opened state (Fig. 4A,E, section d). Amplitude histograms (Fig. 4B–D and F–H) show this increase in open state probability of TRPV1 during pBQ/NAPQI application. The absolute open channel probability reached about 60% while challenged with pBQ, and about 50% with NAPQI. These data strongly indicate that pBQ and NAPQI gate TRPV1 by directly interacting with the channel protein, and not through an indirect mechanism.

### Reducing agents do not reverse pBQ- and NAPQI-induced sensitization of TRPV1

A recent study showed that pBQ modifies thiols of cysteine residues to activate and desensitize TRPA1. Furthermore, chemical modification of cysteines was described as mechanism of TRPV1 sensitization by oxidants^17^. The reducing agent N-acetylcysteine (NAC) has long been used in acetaminophen overdose to limit hepatotoxic effects of reactive metabolites^10,11^. We used NAC as well as the reducing agents dithiothreitol (DTT) and ß-mercaptoethanol to examine whether or not pBQ and NAPQI might interact with cysteines to modify TRPV1. Co-application of NAC (1 mM) with pBQ (Fig. 5A) and NAPQI (Fig. 5B) or DTT (1 mM) with pBQ (Fig. S,6A) and NAPQI (Fig. S6C) completely abolished sensitization of membrane currents mediated by TRPV1. Following wash out of the antioxidants, pBQ and NAPQI immediately sensitized TRPV1 to a similar extent observed in the experiments shown in Fig. 1 (n = 6–7 for each group). In contrast to a sensitizing effect of DTT on TRPV1 described in a previous study^26^, 5 mM DTT applied alone for several minutes did not affect TRPV1-mediated membrane currents (Fig. S,7A, n = 6), nor did NAC (1 mM, Fig. S,7B). Once TRPV1 had been sensitized by pBQ, neither NAC (1 mM, Fig. 5C) nor DTT (5 mM, Fig. S,6B) or ß-mercaptoethanol (1 mM, Fig. S,6E) could reverse these effects (n = 4- 8 for each group). Similar results were obtained for NAPQI-induced sensitization of membrane currents, i.e. these could not be reversed by 1 mM NAC (Fig. 5D) or DTT (Fig. S,6D, n = 6- 7).

### Sensitization of TRPV1 by pBQ and NAPQI involves intracellular cysteines

**Figure 6:**
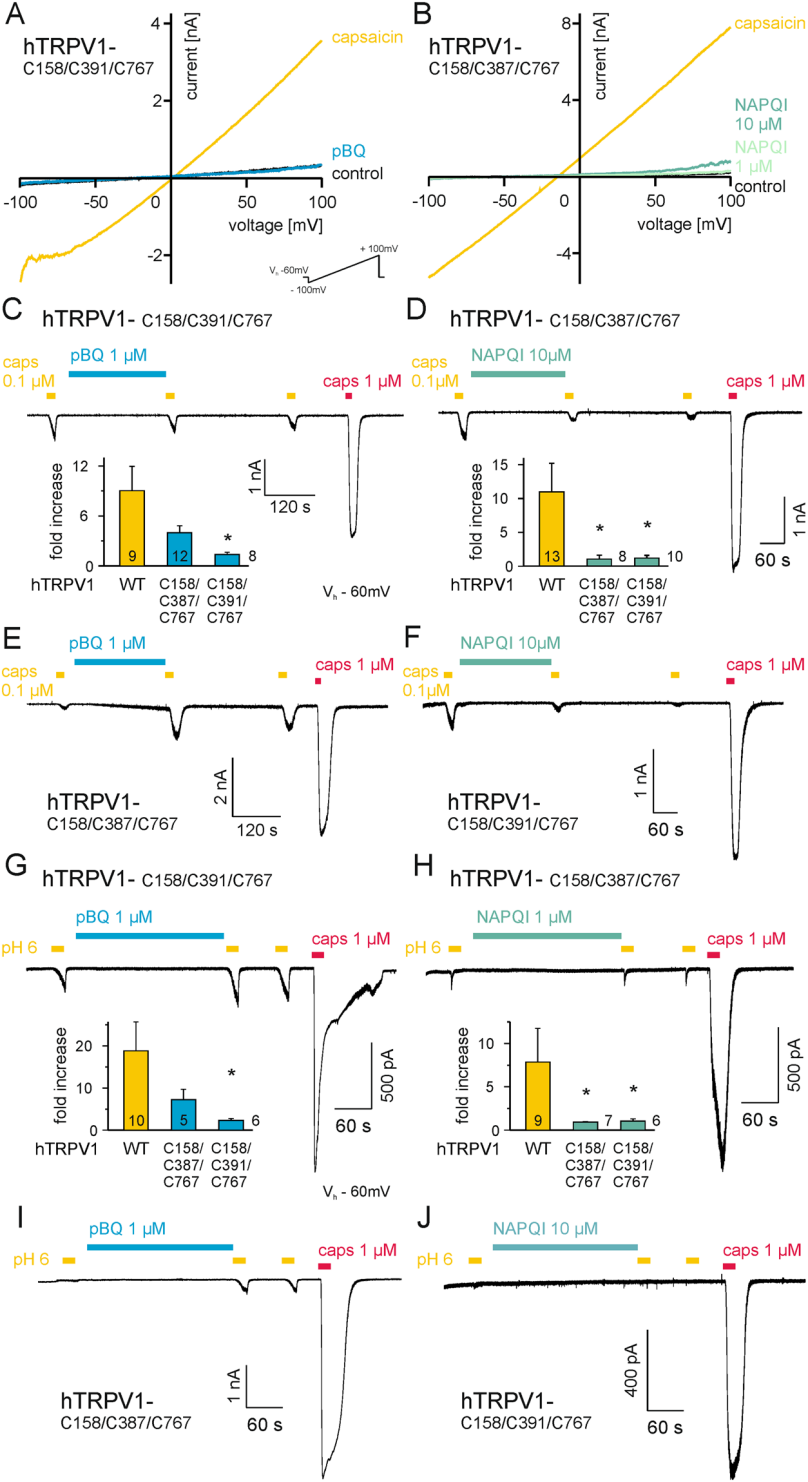
Modulation of cysteines underlies the sensitization of TRPV1 by pBQ and NAPQI. (**A**,**B**) Mutation of internal cysteines in hTRPV1 abolishes sensitization of by both pBQ (G C158A/C391S/C767S-hTRPV1) and NAPQI (H C158A/C387S/C767S-hTRPV1). pBQ and both concentrations of NAPQI were applied for five minutes. These cysteines are also involved in sensitization of capsaicin- (**C**–**F**) and proton-induced (**G**–**J**) inward currents by both metabolites. Representative traces are displayed for pBQ (C and G, C158S/C391S/C767S-hTRPV1; (**E** and **I**) C158A/C387S/C767S-hTRPV1) and NAPQI (**D** and **H**), C158A/C387S/C767S-hTRPV1; F and J, C158S/C391S/C767S-hTRPV1). Bar diagrams show multiples of capsaicin- or proton-induced responses before and after application of pBQ (**C** and **G**) or NAPQI (**D** and **H**) in wild-type and mutant hTRPV1. Sensitization of capsaicin- and proton-induced responses was reduced for pBQ in C158A/C391S/C767S-hTRPV1 (*p ≤ 0.007), and completely lost for NAPQI in both mutants (*p ≤ 0.0009 respectively *p ≤ 0.005).

For rat TRPV1, three extracellular cysteines (C616, C621 and C623) near the pore forming region (marked green in Fig. 5E) were already described to mediate redox-dependent sensitization. Susankova and colleagues generated a single C621G and a triple rTRPV1 mutant (3CYS-rTRPV1)^26^, and we first examined these constructs with pBQ. However, pBQ still activated membrane currents in C621G-rTRV1 (Fig. S,6F) as well as in the triple cysteine rTRPV1 mutant (Fig. 5F, n = 4 each). In hTRPV1, Chuang and Lin^17^ described the four intracellular cysteines C158, C387 and C391 in the N-terminal and C767 in the C-terminal (marked with yellow dots in Fig. 5E) to be prone to chemical modification. By the use of two triple cysteine mutants C158A/C387S/C767S hTRPV1 and C158A/C391S/C767S hTRPV1, the authors proved a role of those cysteines for sensitization of hTRPV1 by oxidants to activation by capsaicin and protons. Therefore, we examined these two triple mutants with pBQ and NAPQI. While 1 µM pBQ induced a 12-fold (±4, mean ± SEM) increase of outwardly rectifying currents on wild-type hTRPV1, the increase was 6.5-fold ( ± 2) on C158A/C387S/C767S-hTRPV1 (p = 0.31, Fig. S8A). On C158A/C391S/C767S-hTRPV1, pBQ failed to significantly potentiate membrane currents (1.4 ± 0.3-fold, p = 0.002, U-tests, n = 5–8, Fig. 6A). 1 µM NAPQI induced a similarly strong potentiation on wild-type hTRPV1 (15.6-fold ± 4). Again, the triple mutants displayed a strongly reduced potentiation, i.e. 1.5-fold ( ± 1) in C158A/C387S/C767S-hTRPV1 (p = 0.006; Figs. 6B and 1.4-fold ( ± 0.4) in C158A/C391S/C767S-hTRPV1 (p = 0.004; Fig. S8B; U-tests, n = 6 for each group).

1 µM pBQ, applied for 4 minutes between the first and a second capsaicin 0.1 µM stimulus evoked a 9 ( ± 3)-fold increase in capsaicin-induced inward currents (Fig. 2A). This potentiation was lost in the C158A/C391S/C767S-hTRPV1 (1 ± 0.3-fold increase; p = 0.001; n = 8; Fig. 6C), but unchanged in the C158A/C387S/C767S-hTRPV1 mutant (4 ± 1-fold increase; p = 0.1; n = 12; Fig. 6E). 10 µM NAPQI potentiated capsaicin-induced inward currents in the wild-type hTRPV1 (11 ± 4-fold increase; Fig. 2B). However, NAPQI did not evoke any potentiation of inward currents evoked by repeated capsaicin stimuli in both triple cysteine mutants (C158A/C391S/C767S-hTRPV1: 1 ± 0.6-fold, C158A/C387S/C767S-hTRPV1: 1 ± 0.4-fold increase; both p = 0.001; n = 8-10; Fig. 6D and F). 1 µM pBQ also induced a strong sensitization of proton-induced (pH 6.0) inward currents in wild-type hTRPV1 (21 ± 6-fold, Fig. 2C), and this potentiation was again almost abolished in C158A/C391S/C767S-hTRPV1 (2.4 ± 0.5-fold, p = 0.007), but unchanged in C158A/C387S/C767S-TRPV1 (7.3 ± 3-fold, p = 0.09, Fig. 6G and I, n = 5–10). Both triple mutations abolished the sensitizing effects of NAPQI on proton-evoked inward currents. While proton-evoked currents of wild-type hTRPV1 were potentiated about 7.9-fold ( ± 3) by NAPQI, this effect was reduced to 0.9-fold ( ± 0.1) on C158A/C387S/C767S-hTRPV1 (p = 0.0009, Fig. 6H) and to 1.1-fold ( ± 0.3) on C158A/C391S/C767S-hTRPV1 (p = 0.005, Fig. 6J, all Mann Whitney U-tests, Bonferroni correction for three groups, n = 6–9).

### pBQ and NAPQI induce an hTRPV1-mediated rise in intracellular calcium

**Figure 7:**
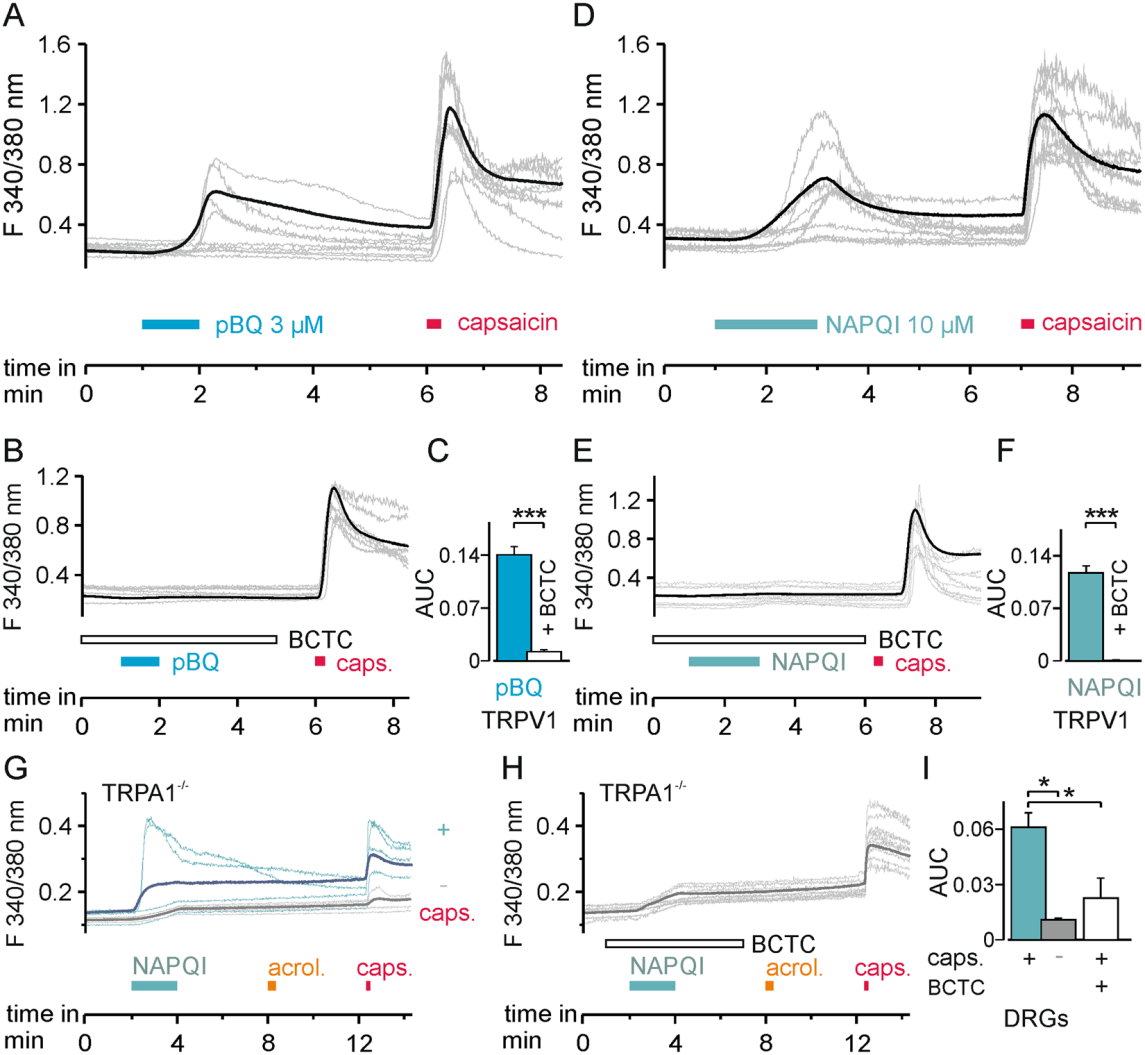
pBQ and NAPQI induce a TRPV1-dependent rise in intracellular calcium. (**A**) In hTRPV1-expressing HEK 293 cells, 3 µM pBQ induced a rise in intracellular calcium in 36% of cells which also responded to capsaicin (n = 392). (**B**) Increase in intracellular calcium by pBQ was inhibited by the TRPV1 blocker BCTC (100 nM; n = 340). Responses are shown as mean of all cells responsive to pBQ (**A**) or capsaicin (**B** bold black trace), and calcium measurements of representative cells (thin grey traces). (**C**) Areas under the curve (AUC) of evoked calcium signals of all cells responding to pBQ: pBQ responses (blue bar) were significantly reduced by the TRPV1 channel blocker BCTC (white bar p < 0.001). (**D**) Similar to pBQ, 10 µM NAPQI evoked increases in intracellular calcium in hTRPV1-expressing cells (59%, n = 282). (**A**) These responses were inhibited by BCTC in 95% of capsaicin-responsive cells (n = 602). Bold black trace (mean) and thin grey traces (representative cells) of calcium measurements. (**F**) AUC of evoked calcium signals of all cells measured responding to NAPQI: NAPQI responses (cyan bar) were significantly reduced by BCTC (white bar; p < 0.001). (**G**) 10 µM NAPQI induced an increase in intracellular calcium in 15% of DRG neurons from TRPA1-knockout mice. Calcium responses of capsaicin-responding (blue, n = 216) and non-responding (gray, n = 166) subgroups of DRG neurons (bold = mean, thin = representative measurements of each group). (**H**) Co-application of the TRPV1 blocker BCTC reduced NAPQI-evoked calcium responses in DRG neurons from TRPA1-knockout mice (bold = mean of capsaicin-sensitive neurons, thin grey traces display representative measurements). (**I**) Comparison of the AUCs of calcium increase of the traces presented in (**G** and **H**). Increases in intracellular calcium following application of NAPQI are significantly inhibited in cells unresponsive to capsaicin (gray bar) or by TRPV1 block by BCTC (white bar; p = 0.00002).

To confirm the results obtained by patch clamp recordings, we used ratiometric calcium imaging on hTRPV1-expressing HEK 293 cells. 3 µM pBQ applied for 60 s evoked an increase in intracellular calcium in 36% of cells also responding to 0.3 µM capsaicin (Fig. 7A and C blue bar, n = 392). According to the notion that this effect is mediated by hTRPV1, this pBQ-induced effects could be abolished by the TRPV1-blocker BCTC (100 nM) which reduced the area under the curve (AUC) of intracellular calcium from 0.12 ± 0.01 in cells responding to pBQ to 0.001 ± 0.01 in the presence of BCTC (Fig. 7B and C white bar, n = 340, ANOVA F(1, 730) = 77.830; p < 0.001; followed by HSD post hoc tests p < 0.001). Similar to pBQ, application of 10 µM NAPQI for 120 s on hTRPV1-expressing HEK 293 cells evoked responses in 59% of capsaicin-sensitive cells (AUC of intracellular calcium 0.14 ± 0.1, Fig. 7D and F cyan bar, n = 282). Again, the TRPV1-blocker BCTC effectively blocked these responses (AUC of intracellular calcium 0.01 ± 0.1, Fig. 7E and F white bar, n = 602, ANOVA F(1,882) = 131.97; p < 0.001; followed by HSD post hoc tests p < 0.001).

### NAPQI, but not pBQ induces TRPV1-mediated calcium responses in DRG neurons

In mouse DRG neurons, activation of TRPA1 by pBQ was reported already at low concentrations (<1 µM)^5^. In accordance to these findings, it was observed that 1 µM pBQ induced a strong increase in intracellular calcium in DRG neurons of wild-type mice which also responded to the TRPA1-agonist acrolein (n = 39, Fig. S9A). In DRG neurons from TRPA1-knockout mice, however, 1 µM pBQ failed to evoke responses (n = 78, Fig. S9B). 10 µM pBQ on the other hand, evoked an increase in intracellular calcium in DRG neurons from mice lacking both TRPA1 and TRPV1 (28%, n = 303, Fig. S9C). Thus, the specificity of pBQ for TRPA1 and possibly TRPV1 in DRG neurons may vanish at concentrations exceeding 10 µM pBQ. In DRG neurons derived from TRPA1-knockout mice, 10 µM NAPQI induced an increase in intracellular calcium in 15% of all examined neurons (AUC of intracellular calcium 0.06 ± 0.01). Notably, this NAPQI-sensitivity was strongly limited to the population of neurons which also responded to 0.3 µM capsaicin, e.g. to cells expressing TRPV1 (n = 372; Fig. 7G, I). Accordingly, BCTC significantly reduced NAPQI-induced increases in intracellular calcium in capsaicin-sensitive TRPA1^−/−^ DRG neurons (AUC of intracellular calcium 0.02 ± 0.01, Fig. 7H and I (white bar), n = 84, ANOVA F (2, 413) = 63.620; p < 0.001; followed by HSD post hoc tests, p < 0.00002).

### Block of TRPV1 does not prevent acetaminophen-induced cell death in mouse hepatocytes

**Figure 8:**
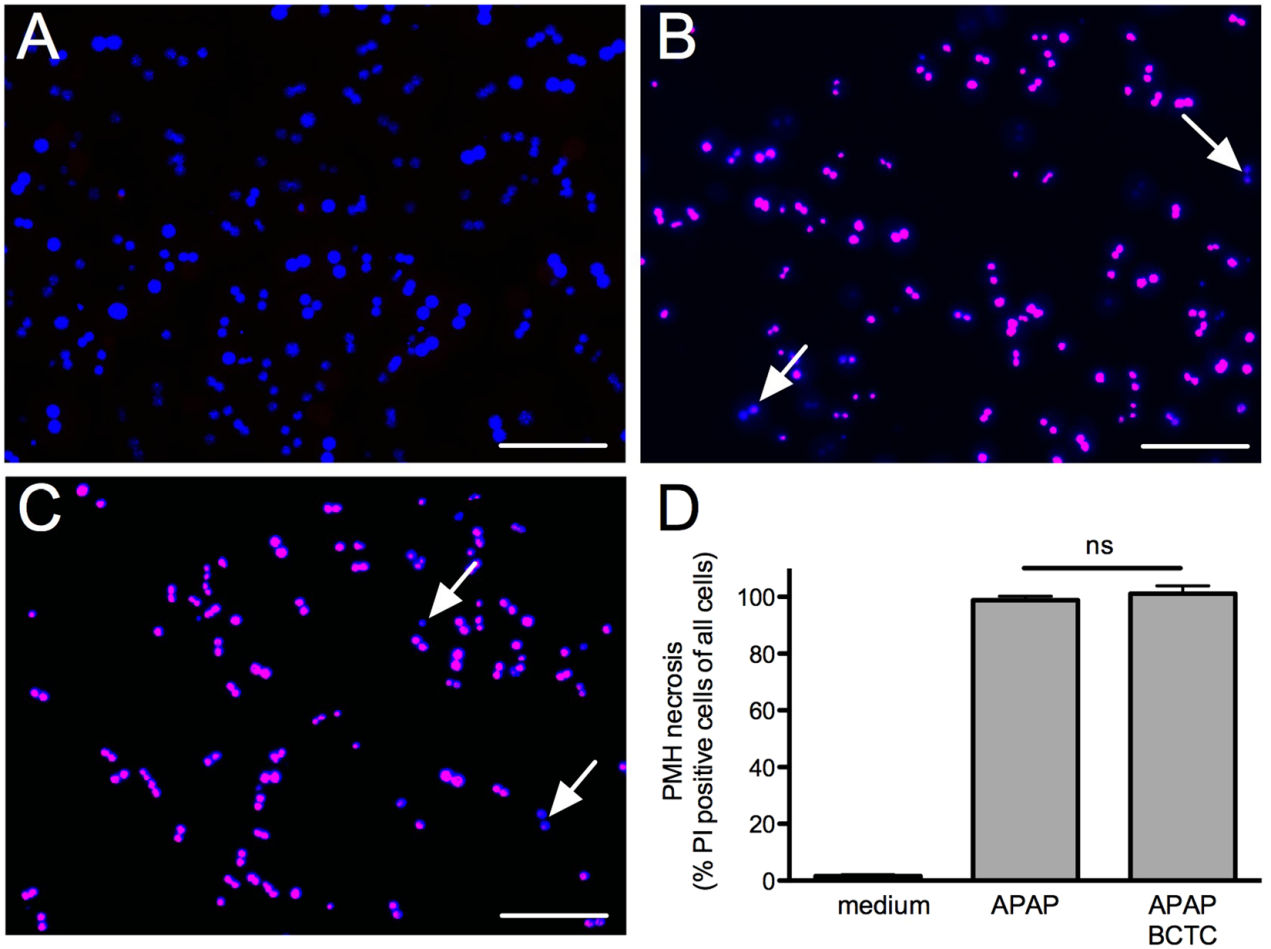
Block of TRPV1 does not inhibit acetaminophen-induced hepatotoxicity. (**A**–**C**) Primary mouse hepatocytes were treated with medium (**A**), 10 mM acetaminophen (**B**) or acetaminophen and 10 µM BCTC (**C**) for 22 h and stained with propidium iodide (PI, red) to visualize necrotic cells and DAPI (blue) for staining the nucleus of all cells. In (**A**) there are only blue colored nuclei visible indicating the absence of necrotic cells. Whereas the treatment with acetaminophen (**B**) even in combination with BCTC (**C**) resulted in almost 100% necrotic cells that are DAPI and PI positive and appeared as purple colored nuclei (arrows point to DAPI positive and PI negative cell nuclei; scale bar = 100 µM). (**D**) Bar graphs indicate percentage of necrotic cells following treatment of mouse hepatocytes with 10 mM acetaminophen, the TRPV1 blocker BCTC at 10 µM is not efficient to reduce or prevent cell death.

Activation of TRPV1 can induce cell death^27^, and NAPQI is regarded to be responsible for acetaminophen-induced hepatotoxicity^11^. Expression of TRPV1 in hepatocytes (Fig. S10) and its relevance in hepatopathology has been demonstrated in previous reports^20,21^. We therefore investigated the involvement of TRPV1 for acetaminophen-induced hepatotoxicity on cultured mouse hepatocytes. Cells were treated with 10 mM acetaminophen for 22 h, which induced cell death in 98% of cells. However, this effect could not be prevented or blunted by co-application of 10 µM BCTC (Fig. 8, n = 3, Kruskal-Wallis ANOVA followed by Dunn’s post hoc test). Applying capsaicin in increasing concentrations (1 µM- 100 µM) for one to two minutes in calcium imaging of Fura-2-AM loaded hepatocyte cultures of mice, we could not detect any increase in intracellular calcium (n ≥ 49 cells, data not shown).

### Injection of NAPQI evokes pain and an increase in cutaneous blood flow in humans

**Figure 9:**
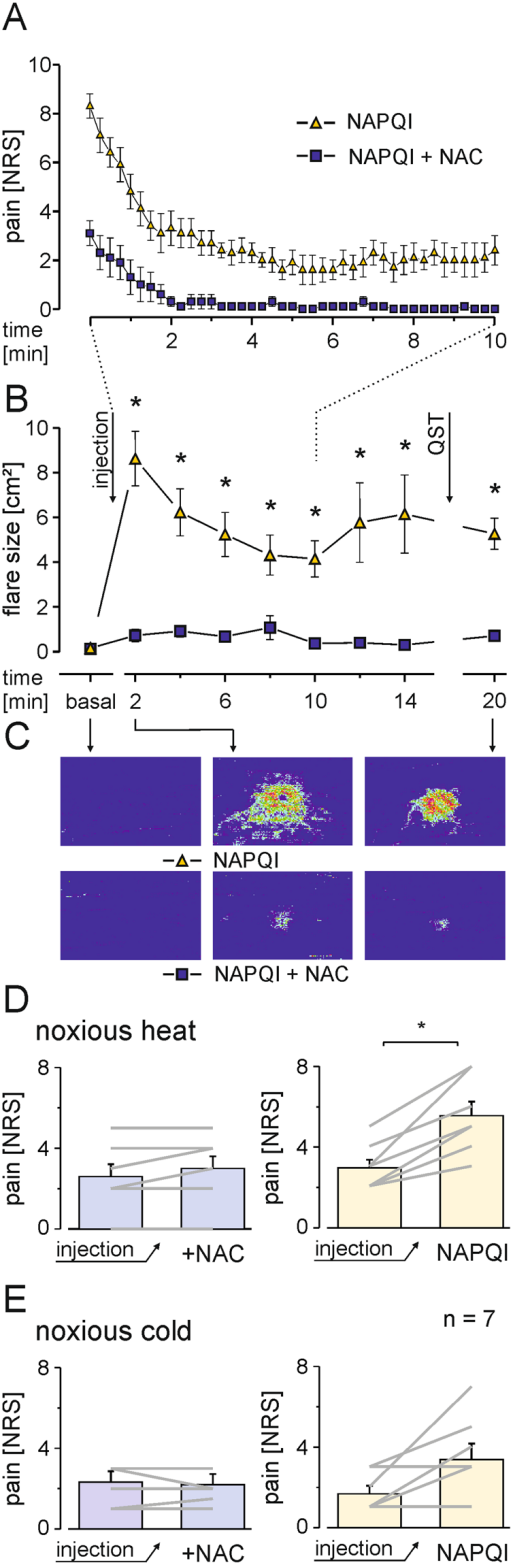
Injection of NAPQI in human skin induces pain and axon-reflex erythema. (**A**) Magnitude and time course of NAPQI-evoked pain in human volunteers after intracutaneous injection to the volar forearm. Pain was rated on a numerical rating scale (NRS) from 0 to 10. With co-application NAC (10 mM), pain following injection of NAPQI was reduced (n = 7, mean ± SEM). (**B**) Laser Doppler scanning to measure superficial blood flow reveals increased flow after NAPQI injection in comparison to NAPQI and NAC (p ≤ 0.011). (**C**) Representative pseudocolor image series of NAPQI ± NAC injections in one volunteer. (**D**) Pain ratings to noxious heat (47 °C, 10 s) increase following intracutaneous injection of NAPQI, but not when co-applied with NAC. Diagrams show responses before after injection of NAPQI alone (right) or in combination with NAC (left) presented as mean ± SEM and responses of all subjects tested (grey lines; *p = 0.018). (**E**) Only in some volunteers NAPQI alone (right), but not in combination with NAC (left) also increased responses to noxious cold (0 °C, 10 s; p = 0.069).

Activation of spinal TRPA1 channels by NAPQI has been proposed as a mechanism for antinociceptive effects of acetaminophen. Nevertheless, activation of TRPA1 and TRPV1 in endings of nociceptive nerve fibers primarily causes pain. The action potentials which follow membrane depolarization by TRP channel activation are not only propagated to the central nervous system leading to the experience of pain, but also cause a release of vasoactive neuropeptides in the periphery. Calcitonin gene-related peptide (CGRP), one of the most potent endogenous vasodilators, is concomitantly released from the activated peptidergic C-fibers as well as from their wide-branching collaterals by antidromic action potential propagation. This so-called “axon-reflex” flare leads to a widespread and sometimes patchy reddening of the skin. We hypothesized that if NAPQI indeed activates human TRPA1 and TRPV1 channels *in vivo*, an injection to human skin should cause pain and axon reflex flare. This hypothesis was tested as proof of concept in human volunteers (n = 7; 3 male and 4 female) by a double-blinded intracutaneous injection of 75 µl of either NAPQI (10 mM) or NAPQI and NAC (10 mM). Indeed, the injection of NAPQI caused an immediate and severe burning pain with a mean maximum pain rating of 8.3 ± 0.5 on a numerical rating scale (NRS from 0- 10). This intense pain was rapidly declining over approximately 4 min to persist on lower levels (NRS 3) for about 15 min (Fig. 9A). The pain quality was described as burning, stinging or stabbing, and was accompanied by slightly itching sensations in all volunteers (Fig. S11). Injection pain of NAPQI was strongly reduced, but not completely abolished when co-applied with NAC. Responses to noxious heat (10 s, 47 °C) were significantly enhanced by injection of NAPQI, (Fig. 9D, p = 0.018), while only four volunteers reported increased ratings to noxious cold (10 s, 0 °C, Fig. 9E, p = 0.069) and mechanical allodynia. When NAC was co-injected, neither allodynia nor changes in responses to noxious heat or cold were observed (n = 7 each; Wilcoxon matched pairs test). In addition to injection pain, NAPQI induced a large axon-reflex erythema visualized by laser Doppler scanning of superficial blood flow of the forearm (Fig. 9B). This erythema was strongly reduced by a co-application of NAPQI and NAC. Notably, a slight and transient increase in cutaneous blood flow caused by the injection needle itself can also not be excluded to add to erythema in both groups (Fig. 9B and C; Fig. S12; repeated measures ANOVA F(9, 4) = 13.796; p = 0.011; following HSD post hoc tests; p ≤ 0.011).

## Discussion

In this paper we show that the acetaminophen metabolites pBQ and NAPQI sensitize and activate TRPV1. Using site directed mutagenesis and antioxidants we could identify chemical modification of intracellular cysteines by pBQ and NAPQI as the main gating mechanism of TRPV1. NAPQI and pBQ also sensitized heat-induced currents in mouse DRG neurons, thus confirming a similar effect on TRPV1 in native neurons. In mouse hepatocytes however, TRPV1 did not seem to be involved in acetaminophen-induced hepatotoxicity. Finally, NAPQI induced injection pain and subsequent heat hyperalgesia in human volunteers. This finding argues for activation of TRPV1 by NAPQI in human nociceptors *in vivo*.

Besides several suggested spinal and supraspinal mechanisms of action^3,4^, the analgesic effects of acetaminophen have recently been attributed to a spinal metabolism to pBQ and NAPQI and a subsequent activation of TRPA1^5^. Similar to TRPA1, TRPV1 is expressed in sensory neurons and in different regions throughout the central nervous system^28–30^. In our experiments, both pBQ and NAPQI showed characteristic properties of TRPV1 agonists and they also sensitized the channel to capsaicin, protons and heat. Several other TRPA1 agonists have also been shown to sensitize and activate TRPV1 at high concentrations^16^, and this may be due to the fact that both channels are prone to chemical modification by oxidants. While three N-terminal cysteines have been described to confer the sensitivity of TRPA1 to reactive substances^8,31^, both N- and C-terminal cysteines have been described to be important for sensitization of TRPV1 by oxidation^17^. Both pBQ and NAPQI are highly reactive and can modify several amino acids including cysteines^12,32^. Indeed, our data revealed that both metabolites sensitize TRPV1 by modifying critical cysteines. We could also exclude an effect of pBQ at the capsaicin-binding site, and pBQ did not seem to interfere with PKC-dependent phosphorylation of TRPV1. Besides acetaminophen, other frequently used analgesics such as dipyrone have been suggested to involve TRP channels to induce analgesia^33^. Activation of TRPV1 in descending inhibitory pain pathways has been suggested as analgesic mechanism even for acetaminophen^34,35^. While inhibition of action potentials due to a reduction of Na^+^ and Ca^2+^ currents following activation of TRPA1 by NAPQI have been proposed as a mechanism of how spinal activation of TRPA1 channels might contribute to analgesia^5^, further mechanisms remain elusive. However, presynaptic inhibition by activation of TRPA1 by other agonists could be confirmed in a more recent *in vivo* electrophysiological study^36^. Similar properties have also been suggested for TRPV1 channels expressed along peripheral axons and in the spinal cord, as activation of TRPV1 is followed by a prolonged inhibition of voltage-gated Na^+^ and Ca^2+^ channels^37,38]^. In addition, desensitization of TRP channels following activation have also been proposed^39^.

Although effects of pBQ in transfected HEK 293 cells were clearly dependent on TRPV1, results in mouse DRG neurons were more difficult to interpret as we could not show a specific activation of TRPV1 by 1 µM pBQ, and an unspecific effect 10 µM. While TRPA1 is the channel being most potently activated by pBQ in mouse DRG neurons, higher concentrations of pBQ clearly evoked responses in TRPA1/TRPV1 double knockout mice. Nevertheless, it seems obvious that ion channel activation or modification by reactive metabolites is not restricted to TRPA1 and TRPV1 in sensory neurons. For example, both TRPM2 and TRPV4 have been shown to be sensitized and activated by H_2_O_2_ or even NAPQI, and both channels are expressed in DRG neurons^40–43^. When examining neurons from TRPA1-knockout mice in presence of the TRPV1-blocker BCTC however, we could isolate a TRPV1-expressing subgroup of DRG neurons responding to NAPQI. Nevertheless, both pBQ and NAPQI strongly sensitized heat-induced currents in DRG neurons independent of TRPA1. Using capsaicin to select TRPV1-positive small DRG neurons and heat stimuli below 45 °C will most probably exclude effects mediated by TRPV2, as this heat responsive ion channel requires higher temperatures for its activation and DRG neurons barely co-express TRPV1 and TRPV2^28^.

Besides TRPA1 as a possible spinal mechanism, it was already postulated that TRPV1 expressed in the CNS critically contributes to acetaminophen-induced analgesia by being activated by the metabolite AM404^35,44,45^. Cytochrome P450 isoforms metabolizing acetaminophen to NAPQI are also present in the brain, and apart from analgesia NAPQI has been accused of having neurotoxic in the CNS effects by causing oxidative stress^46^. Importantly, activation of TRPV1 has been shown to directly promote cytotoxicity and cell death^27,47,48^. While cell death induced by stimulation of TRPV1 has been suggested as a possible therapeutic mechanism even in the brain^49^, it might as well contribute to neuronal degeneration. Indeed, experimental studies on rodents identified neurotoxic effects in the CNS even by recommended and thus clinically used dosages of acetaminophen^50^. While the exact mechanisms and the clinical relevance of these findings are yet to be determined, our data suggest that TRPV1 might be a relevant mechanism. Although acetaminophen is considered to be relatively safe at therapeutic doses, hepatic failure due to overdosing has been an important and clinically relevant issue. NAPQI-induced oxidative stress seems to be one of the key mechanisms for the hepatotoxicity of acetaminophen^11^. Furthermore, high doses of NAC are effective as an antidote when administered at an early stage following intoxication^10^. In our experiments, pBQ and NAPQI-induced activation of TRPV1 could not be reversed by NAC and other reducing agents. However, co-application of NAC and one of the metabolites completely prevented any sensitizing effect on TRPV1, thus suggesting a scavenging mechanism of NAC. A similar role of NAC seems to apply for treatment of hepatotoxicity when reactive metabolites might be directly bound to NAC or are indirectly detoxified by restored levels of glutathione. Although NAPQI and pBQ are excessively produced in the liver following acetaminophen overdosing, we did not observe a protective effect of the TRPV1-blocker BCTC in a cell culture model of acetaminophen-induced hepatotoxicity. Thus, although activation of TRPV1 in hepatocytes is likely to occur when acetaminophen is overdosed, our *in vitro* data do not suggest that TRPV1 contributes to hepatotoxicity in mice.

Systemic application of TRPV1 agonists induces hypothermia, and the development of TRPV1 antagonists for pain treatment has significantly been complicated by the fact that many of the drugs in development cause hyperthermia^51,52^. Control of body temperature involves visceral afferent input of TRPV1-expressing fibers. Therefore, sensitizing TRPV1-receptors to heat by acetaminophen metabolites, which we could show in different experiments, could hypothetically increase this afferent input and initiate a negative feedback mechanism to lower body temperature. However, recent studies could not prove any involvement of TRPA1 or TRPV1 in the antipyretic effects of acetaminophen^53^. Importantly though, the induction of fever in TRPV1-knockout mice is difficult as responses to pyrogens are strongly attenuated^54^.

TRPV1 and TRPA1 are primarily regarded as nociceptive channels, i.e. their activation in peripheral nerve endings promotes the sensation of pain. Activation of TRPA1 and TRPV1 has also been suggested to underlie the injection pain evoked by substances like lidocaine and propofol^55,56^. We observed pronounced NAPQI-evoked injection pain and concomitant axon reflex erythema in human volunteers, indicating that human nociceptive C-fibers are activated and vasoactive neuropeptides such as calcitonin gene-related peptide are released^57^. Although we cannot distinguish between the TRP channels activated in these experiments, the severe heat hyperalgesia argues for an involvement of TRPV1^58^. As acetaminophen clearly never induces pain following systemic administration, it seems obvious that NAPQI does not reach peripheral nerve endings at concentrations sufficient to activate TRP channels. Thus our data rather speak in favor of spinal or central sites for NAPQI-derived analgesic effects of acetaminophen. Indeed, intraplantar injections of APAP in mice did neither cause analgesic nor algesic effects^5^. Furthermore, 50 mM acetaminophen injected intracutaneously in human volunteers was neither painful nor influenced quantitative sensory testings^59^. Both findings argue against peripheral nerve endings as important site of acetaminophen-induced effects and against a relevant metabolism of acetaminophen into NAPQI and pBQ in the skin.

Taken together, acetaminophen is metabolized to different reactive siblings at peripheral and central sites. Activation of TRP channels by these metabolites may have both beneficial and harmful effects depending on the site of receptor activation. Therefore, it is not only important to investigate and understand which metabolites are generated, but also at which sites they accumulate. In this study we show that the reactive metabolites pBQ and NAPQI cause profound sensitization and activation of TRPV1. This adds to the complex picture of mechanisms of how acetaminophen could induce its well appreciated analgesic and antipyretic effects, but also unwanted and harmful cytotoxic effects including hepatotoxicity. Further research is needed to unravel the role of TRP channels in the complex pharmacology of acetaminophen.

## Methods

### Chemicals

Chemicals were purchased and solved as follows. TRPV1 channel blocker BCTC and ionomycin (both 10 mM in DMSO) were from Tocris (Bio-Techne, Wiesbaden-Nordenstadt, Germany). pBQ (100 mM stock solution in DMSO) and DTT (100 mM stock solution in external solution) were from Sigma-Aldrich (Taufkirchen, Germany). NAPQI was purchased from Dalton Pharma Services (Toronto ON, Canada) and dissolved in DMSO. 250 mM stock solutions were stored at −80 °C and diluted immediately prior to use.

### Plasma concentration of reactive acetaminophen metabolites

Due to the instability of NAPQI in water, its stable cysteine conjugate (L-cysteinyl-acetaminophen) in plasma has been measured and shown to reach 5% of APAP plasma levels^60–62^. Plasma APAP concentrations range from 120-300 µM following oral or i.v. administration of 1 g APAP and similarly 60–100 µM APAP can be detected in the cerebrospinal fluid^63,64^. In acetaminophen overdose L-cysteinyl-APAP plasma concentrations of 5–10 µM have been directly measured in several studies^65,66^, but L-cysteinyl-APAP could also be measured by mass spectrometry in the spinal cord of mice following therapeutic APAP administration in mice^5^. Given the fact that NAPQI is instable in water, local concentrations in tissues metabolizing acetaminophen may also exceed the levels of L-cysteinyl-APAP which have been measured in plasma. pBQ is derived from acetaminophen due to metabolism by cytochrome P450 and COX enzymes which are expressed in the liver but also in the upper lamina of the spinal cord, where TRPV1 is also expressed^5,67–69^. As it is considerably more stable than NAPQI, it is more applicable to experimental investigation.

### Cell culture

For experiments on the wild-type human (h) TRPV1 channel a TRPV1-expressing HEK cell line was used which was a kind gift from Dr. Peter Zygmunt (Clinical Chemistry & Pharmacology, Department of Laboratory Medicine, Lund University Hospital, Lund, Sweden). Otherwise, HEK 293 T cells were transfected with plasmids of rat or rabbit TRPV1, their chimeras or several mutants of mouse and human TRPV1 (1 µg each) using Nanofectin (PAA, Pasching, Austria). cDNA of mouse TRPV1 mutants lacking cysteines in the extracellular domain of TRPV1 (TRPV1 C621G and TRPV1 C616G/C621G/C634G) were a kind gift of Dr. Viktorie Vlachova (Department of Cellular Neurophysiology, Institute of Physiology of the Czech Academy of Sciences, Prague, Czech Republic). Triple mutants lacking cysteines relevant for sensitization of hTRPV1 by oxidation were generated analog to the findings of Chuang and Lin^17^ (TRPV1 C158A, C387S, C767S or TRPV1 C158A, C391S, C767S) by site directed mutagenesis of TRPV1 cDNA using the quikchange lightning site-directed mutagensis kit (Agilent, Waldbronn, Germany) according to the instructions of the manufacturer. All mutants were subsequently sequenced to verify intended amino acid exchanges, and to exclude further channel mutation. Approximately 24 h following transfection, cells were split and seeded for patch clamp or calcium imaging experiments.

Culturing of DRG neurons from C57Bl/6, congenic TRPA1 as well as TRPV1/TRPA1 knockout mice was performed as described previously^70^. Briefly, mice were deeply anaesthetized by isoflurane inhalation, sacrificed by decapitation, and DRGs from all levels were excised and transferred to Dulbecco’s modified Eagle’s medium (DMEM). Following treatment with DMEM containing 1 mg/ml collagenase and 0.5 mg/ml protease for 45 min (both from Sigma-Aldrich, Munich, Germany), ganglia were dissociated using a fire-polished, silicone-coated Pasteur pipette. Isolated cells were transferred onto poly-L-lysine-coated (0.1 mg/ml, Sigma Aldrich) coverslips and cultured in TNB 100 medium supplemented with TNB 100 lipid protein complex, penicillin/streptomycin (100 U/ml) (all from Biochrom, Berlin, Germany), and mouse NGF (100 ng/ml, Almone Laboratories, Tel Aviv, Israel) at 37 °C and 5% CO_2_. Cells were used for experiments within 24 h after plating. TRPA1^−/−^ and TRPV1^−/−^ TRPA1^−/−^ adult mice of both genders were donated by Dr. Peter Reeh (Institute of Physiology and Pathophysiology, University of Erlangen-Nuremberg, Erlangen, Germany). The procedure of DRG neuron isolation was performed in accordance to the regulations and approval of the animal protection authorities (local district government, Hannover, Germany).

### Hepatocyte toxicity assay

The experimental procedures were carried out in compliance with the guidelines for the welfare of experimental animals of the Federal Republic of Germany. The experimental protocols were reviewed and approved by the local District Government of Lower Saxony (33.12-42502-04-16/2269). For primary hepatocyte cultures of mice hepatocytes were isolated from three additional isoflurane-anesthetized adult 10–16-week old male C57BL/6 mice (28–35 g) using a modified two-step hepatic portal vein perfusion method originally described by Klaunig and Zhang^71,72^. After midline incision the portal hepatic vein was cannulated with a 23 G butterfly infusion set (Valu-Set^TM^, Becton Dickinson, Bedford, MA), the inferior vena cava was incised and the liver perfused with 37 °C warmed HBSS without Ca^2+^ and Mg^2+^ containing 0.5 mM EGTA for 10 min with a flow rate of 8 ml/min. *In situ* liver digestion was continued with perfusion of 37 °C warmed DMEM low glucose medium supplemented with 100 U/ml Penicillin and 0.1 mg/ml Streptomycin (Pen/Strep), 15 mM HEPES and 2 mg/ml collagenase-II (Worthington Biochemical, Lakewood, NJ) for another 10 min at 8 ml/min flow rate. The liver was excised, transferred to DMEM medium with collagenase-II and liver sacs were disrupted to release hepatocytes after mechanic agitation. The liver cell suspension was filtered through cell strainers (70 µm, Becton Dickinson, Bedford, MA), centrifuged for 2 min at 50 x g and cell viability determined by trypan blue exclusion. A purity of < 95% and a yield of 30–80 × 10^6^ hepatocytes were achieved. After adhesion of freshly isolated hepatocytes (350.000/well) to collagen-I coated 6-well plates in low glucose DMEM medium supplemented with Pen/Strep and 10% fetal calf serum for 4 h, medium was changed to DMEM without serum and 10 mM acetaminophen or 10 mM acetaminophen and 10 µM BCTC were applied for 22 h at 37 °C in humidified atmosphere containing 5% CO_2_. Necrotic hepatocytes were identified by staining with 10 µg/ml propidium iodide (PI) in PBS, fixation with 4% paraformaldehyde and counterstaining of all cell nuclei with 1 mg/ml DAPI for 15 min at 37 °C each. Five randomly selected high power fields were documented with 100x magnification on an inverted fluorescence microscope (IX81, Olympus, Hamburg, Germany) using Q-Capture Pro7 software (QImaging, Surrey, Canada) and the number of PI and DAPI positive cells were determined using ImageJ version 1.50 g (NIH, USA). Expression of TRPV1 in mouse hepatocyte cultures was confirmed by rtPCR (TRPV1 (NM_001001445.2) forward: GATGACTTCCGGTGGTGCTT; reverse: CCTATCTCGAGTGCTTGCGT) and Western blot using rabbit anti human TRPV1 AK (PAI-748, Thermo Fischer, 1:100) and goat anti rabbit secondary antibody conjugated to HRP (R1364HRP, Acris, 1:5000; Fig. S10).

### Patch Clamp

Whole-cell voltage-clamp was performed on transiently and stably TRPV1- or TRPV1 mutant channel-expressing HEK 293 T cells. Membrane currents were acquired using an EPC10 USB HEKA amplifier (HEKA Elektronik, Lamprecht, Germany), low-passed at 1 kHz, and sampled at 2 to 10 kHz. Electrodes were pulled from borosilicate glass tubes (TW150F-3; World Precision Instruments, Berlin, Germany) to give a resistance of 2.0–5.0 MΩ. The standard external solution was used if not otherwise noted and contained (in mM) NaCl 140, KCl 5, MgCl_2_ 2, EGTA 5, HEPES 10 and glucose 10 (pH 7.4 was adjusted with tetramethylammonium hydroxide or NaOH for single channel recordings and measurements of DRGs). The internal solution contained (in mM) KCl 140, MgCl_2_ 2, EGTA 5 and HEPES 10 (pH 7.4 was adjusted with KOH). If not otherwise noted, cells were held at −60 mV. For current-voltage curves, currents were measured during 500 ms long voltage ramps from −100 to + 100 mV. Single channel recordings were performed using the inside-out patch clamp configuration with internal solution in the bath and external solution in the pipette. Pipettes were pulled from borosilicate glass with a resistance of 10–30 MΩ and were covered with Sigma Cote ® (Sigma-Aldrich, Munich, Germany) to reduce their capacitance. Current traces were low-pass filtered using an analogue 1 kHz eight-pole Bessel filter and subsequently digitally filtered at 300 Hz. Sampling rate was 10 kHz (100 µs). Amplitudes histograms were fitted by Gaussian distributions. If not otherwise noted, all experiments were performed at room temperature. Solutions were applied with a gravity-driven PTFE/glass multi-barrel perfusion system. To apply heat ramps, the Patchmaster software (HEKA Elektronik) was used to pass current to an insulated copper wire coiled around the capillary tip of the common outlet of the perfusion system heating the solutions from room temperature to 45 °C within 5 s. The temperature of the superfusing solution was measured using a miniature thermocouple fixed at the orifice of the capillary tip placed close to the cells under investigation^73^. Data acquisition and off-line analysis were performed by a combination of Patchmaster/Fitmaster software (HEKA Elektronik), Clampex/Clampfit 10.5 software (Axon, Molecular Devices, Sunnyvale CA, U.S.A) and Origin 8.5.1 (Origin Lab, Northampton, MA, U.S.A).

### Ratiometric [Ca^2+^]_i_ measurements

Cells were stained by 3 µM Fura-2-AM and 0.01% pluronic F-127 (both from Biotium Inc., Fremont CA, USA) for about 45 min. Following wash out to allow Fura-2-AM deesterification, coverslips were mounted on an inverse microscope with a 20x objective (Axio observer D1, Zeiss). Cells were constantly superfused with extracellular solution (in mM: NaCl 145, KCl 5, CaCl_2_ 1.25, MgCl_2_ 1, Glucose 10, Hepes 10) using a software controlled 7-channel common-outlet superfusion system. Fura-2 was excited using a microscope light source and an LEP filter wheel (Ludl electronic producs Ltd.) to switch between 340 and 380 nm wavelengths. Images were exposed for 20 and 10 ms respectively and acquired at a rate of 1 Hz with a CCD camera (Cool SNAP EZ, Photometrics). Data were recorded using VisiView 2.1.1 software (all from Visitron Systems GmbH, Puchheim, Germany). Background fluorescence was subtracted before calculation of ratios. A 60 mM potassium stimulus was applied as a control at the end of each experiment for DRG neurons and 5 µM ionomycin was used in HEK293 cells. Cells which did not respond to potassium/ionomycin or showed no functional expression of TRPV1 on capsaicin application in experiments on hTRPV1-transfected HEK293 cells were excluded from the analysis. Averaged results are reported as means ( ± SEM) of area under the curve (delta ratio F340/380 nm) for regions of interest adapted to the cells.

### Psychophysics

Studies on human volunteers were limited to some of the authors and to employees of the department of Anaesthesiology and Intensive Care Medicine of Hannover Medical School. Experimental procedures were approved to fulfil the requirements of the Declaration of Helsinki by the ethics committee of Hannover Medical School (no. 6823). All subjects gave their informed consent after consideration following detailed information on the planned study. Exclusion criteria were defined as follows: acute or chronic disease (physical or mental), allergies, (possibility of) pregnancy, lactation period, taking any medication. All of the seven volunteers could be included in the study. Sample sizes were based on previous experience^31,74^ and elements of the human subjects data have been part of another study^59^. Informed consent contained the following termination criterion: if procedures were experienced as too unpleasant or if the general wish to interrupt the study at any time during the testing was stated, this would lead to the immediate termination of the study without the necessity to give any reasons. None of the seven volunteers made use of it. For the present study either 10 mM NAPQI or NAPQI and 10 mM N-acetylcysteine (NAC) were intradermally injected in 75 μl volume to the volar forearm of the volunteer (n* = *7 each; 3 male and 4 female) in a double-blinded manner. Pain and itch were assessed on a numerical rating scale (NRS), ranging from 0 (no sensation) to 10 (maximum pain/itch imaginable) in 15 s intervals for a period of 10 min. For quantitative sensory testing (QST) numerical ratings (0–10) to a 10 s-lasting heat stimulus of 47 °C or to a 10 s-lasting cold stimulus of 0 °C, applied by 0.8 cm in diameter metal rods heated by a water bath or cooled with ice, were compared before and after application of substances. The skin was tested for dynamic mechanical allodynia by stroking with a fine brush. A laser Doppler imager (moorLDI2-IR, Moor, London, UK) was used to record changes in superficial blood flow. Two baseline scans of 0.6 mm spatial resolution were taken, followed by 7 scans every 2 min starting right after the injections and a final one after 20 min. The areas of superficial vasodilation were analyzed with moorLDI software 5.3 and defined as pixels in which intensity exceeded the mean of basal values plus two standard deviations.

### Statistical analysis

All data are presented as mean ± SEM. Statistical analysis was performed using Statistica 7.1 (Stat soft Inc., Tulsa, USA) or GraphPad Prism 5 (GraphPad Software Inc., La Jolla, USA). Comparisons between two groups with small sample sizes (n ≥ 5) were performed by non-parametric testing with the Wilcoxon matched pair test for paired or the Mann-Whitney U-test for unpaired data. For more than two groups Bonferroni correction was used to adapt p levels. For comparison of the three groups of the hepatocyte toxicity assay a Kruskal Wallis ANOVA was used followed by Dunn’s post hoc test. Analysis of laser Doppler imaging data resulted in image series of seven subjects according to the two treatment groups (NAPQI ± NAC). They were therefore compared by repeated measures ANOVA followed by Tukey HSD post hoc test. Comparisons within the same subjects, namely heat and cold-evoked pain ratings before and after intracutaneous injection of reactive acetaminophen metabolites, were tested using Wilcoxon matched pairs test. Significance was assumed for p < 0.05.

### Data availability

The datasets generated and analysed during the current study are available from the corresponding author on reasonable request.

## Electronic supplementary material

Supplementary Information
