# Supplementary material for: Reactive metabolites of acetaminophen activate and sensitize the capsaicin receptor TRPV1
Source: Sci Rep. 2017 Oct 6;7:12775. doi: 10.1038/s41598-017-13054-3 (PMC5630573; doi:10.1038/s41598-017-13054-3)
Supplement: Supplementary file 1 — Supplementary Information [file 41598_2017_13054_MOESM1_ESM.pdf]

Reactive metabolites of acetaminophen activate and sensitize the capsaicin receptor TRPV1

Mirjam J Eberhardt [a], Florian Schillers [a], Esther M Eberhardt [b], Linus Risser [a], Jeanne de la Roche [c], Christine Herzog [a], Frank Echtermeyer [a], Andreas Leffler [a]*.

Supplementary Information

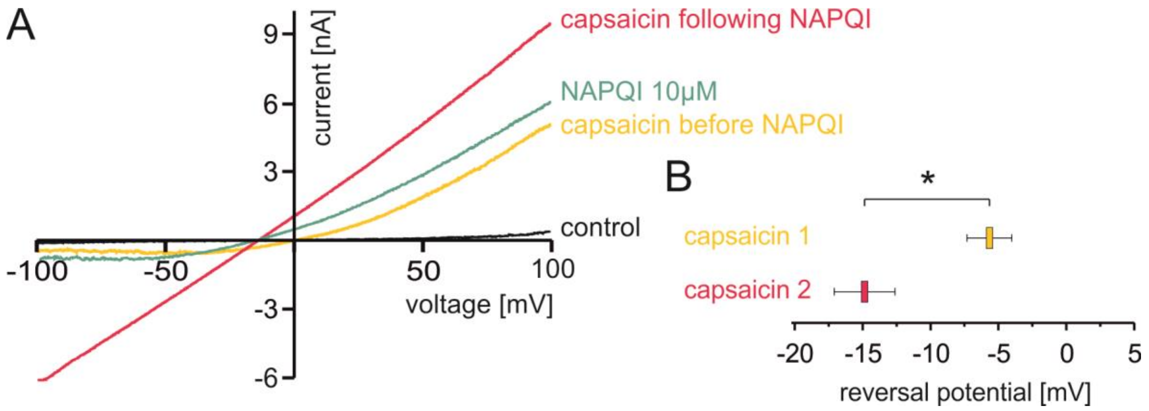

Supplemental Figure 1. Application of NAPQI leads to altered sodium permeability in hTRPV1. A NAPQI 10 µM (3min) was applied following a capsaicin 0.1µM stimulus using patch clamp solutions containing sodium as the sole cation (internal: NaCl 140mM, EGTA 5mM, HEPES 5mM; external: NaCl 140mM, glucose 10mM, HEPES 10mM; pH was adjusted to 7.4 with NaOH). Ramp currents in the presence of a second capsaicin 0.1 µM stimulus showed a leftward shift, as did the reversal potential (**B**) indicating a change in sodium permeability (p = 0.02, n = 7; Wilcoxon matched pairs test).

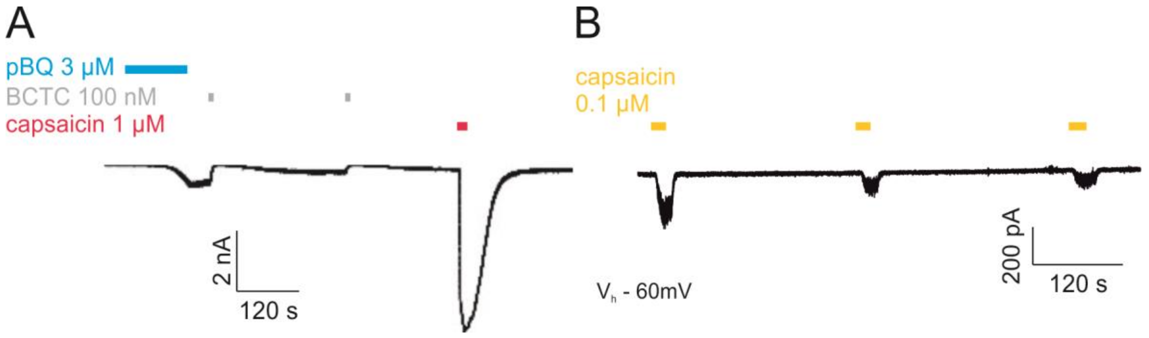

Supplemental Figure 2. A. 3 µM pBQ evokes robust inward currents in hTRPV1-expressing cells held at -60 mV, which can only transiently be blocked by BCTC. B inward currents evoked by repeated capsaicin stimuli (0.1 µM, 20 s) in hTRPV1expressing cells show strong desensitization.

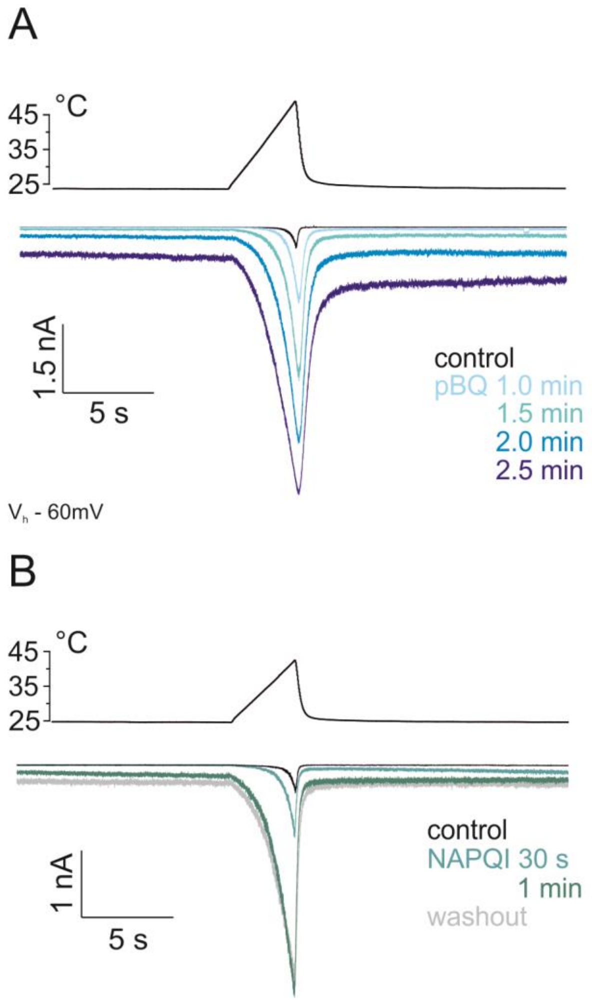

Supplemental Figure 3. pBQ shifts temperature threshold of heat evoked currents in hTRPV1 to lower temperatures. Overlay of all heat-induced currents of one representative recording in an hTRPV1 expressing cell. Note that with duration of pBQ (1 µM) (**A**) and NAPQI (10 µM) (**B**) application, temperature thresholds to evoke an inward current is continuously shifted to lower temperatures.

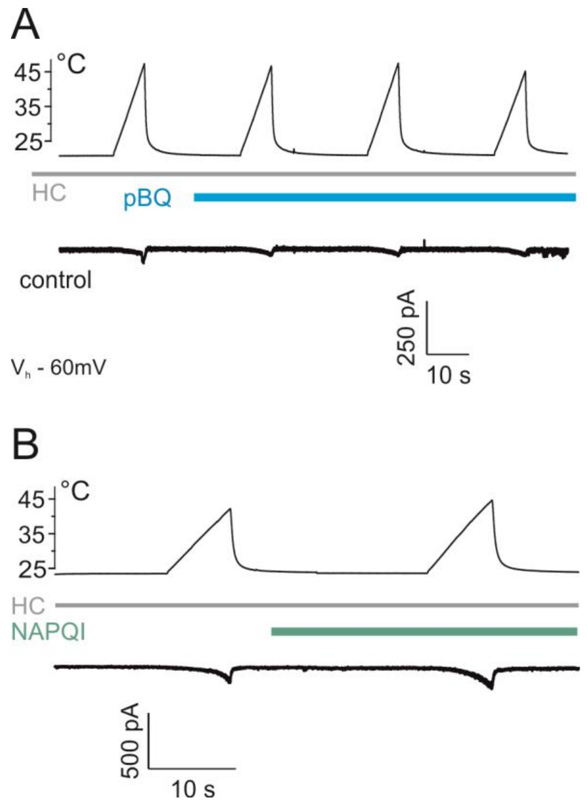

Supplemental Figure 4. pBQ and NAPQI are ineffective in small capsaicin-negative DRG neurons. TRPV1 mediates low threshold (~ 45°C) heat-induced currents in small DRG neurons (Caterina et al., 2000). pBQ (10µM) effectively sensitized heat-induced inward currents within 30s of application (Fig 2 H) in all capsaicin-positive neurons measured. (**A**) When pBQ was applied to small capsaicin-negative DRG neurons which were challenged by heat ramps (up to 45 °C), no currents were evoked even if pBQ was applied for two minutes (n = 2). Similarly, in contrast to experiments performed in capsaicin-positive neurons (Fig. 2J), NAPQI (10µM) had no effect in capsaicin-negative DRG neurons (**B** shows one of three measured neurons). TRPA1 was blocked in these experiments by the channel blocker HC030031.

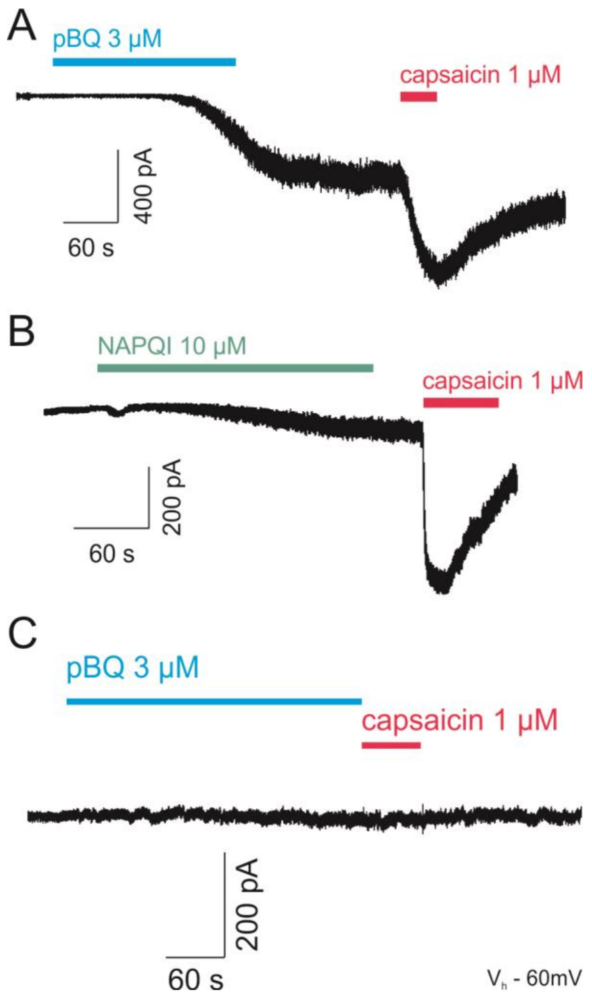

Supplemental Figure 5. Inside-out macro patches: pBQ (3 µM, **A**) and NAPQI (10 µM, **B**) evoked robust currents in inside out membrane patches of hTRPV1-expressing HEK 293 cells. Neither pBQ 3 µM, nor capsaicin 1 µM evokes any currents in inside-out macro patches of untransfected HEK 293 cells (**C**).

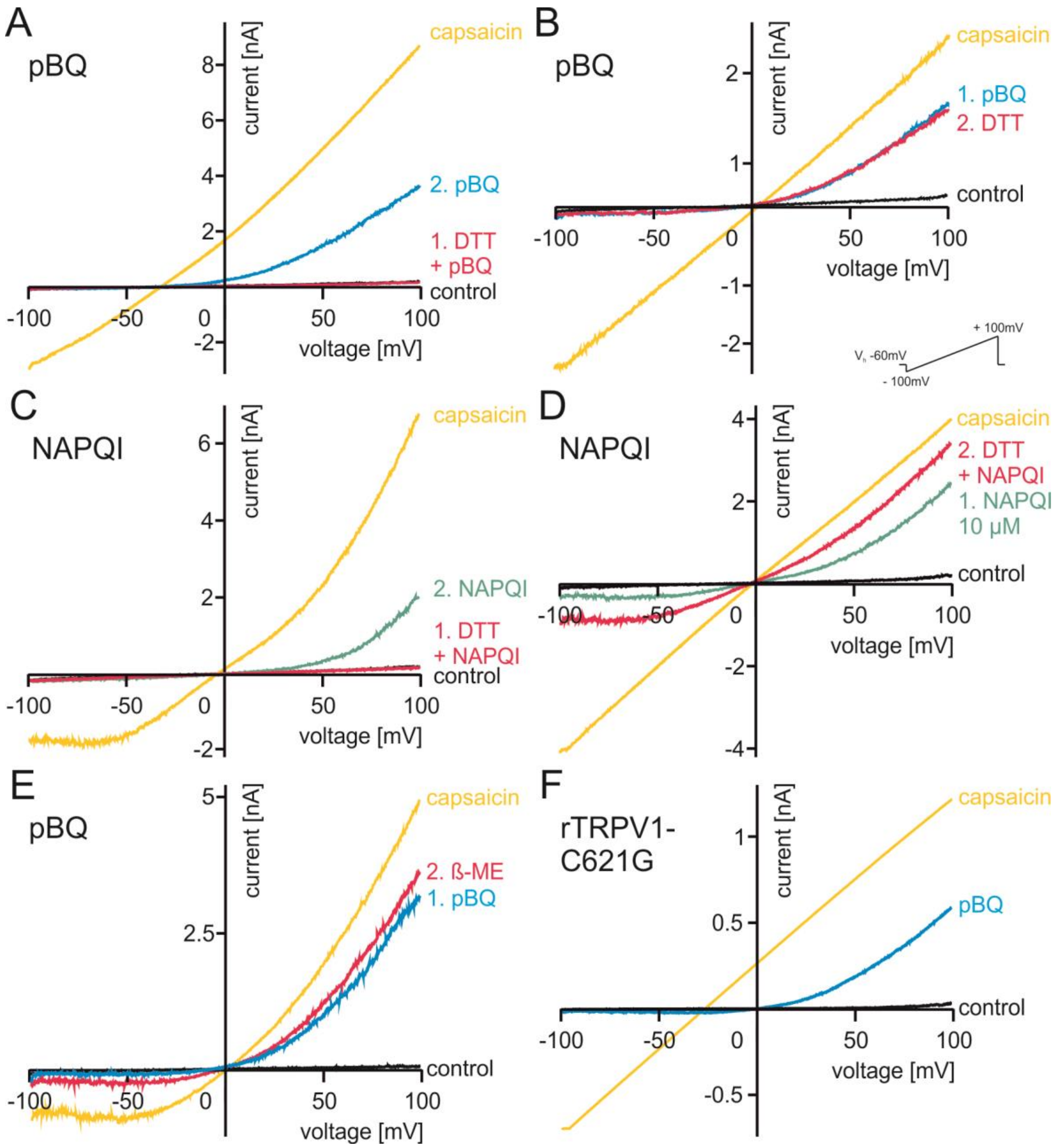

Supplemental Figure 6. A-D. Co-application of dithiothreitol (DTT, 5 mM, at least 4min) prevents sensitization of ramp currents in hTRPV1 by pBQ (**A**) and NAPQI (**C**), while a second application of these reactive acetaminophen metabolites alone for 1.5 – 3minutes again sensitizes hTRPV1. If it is applied once the ramp currents have been sensitized, DTT (5mM, ≥4 min) does not reverse the effects of pBQ (1µM, 3min **B**) or NAPQI (10µM, 3min **D**). E. ß-mercaptoethanol (1 mM, 5min) is also not able to reverse sensitization of TRPV1 once pBQ-induced increase of voltage ramp-induced currents has been fully established. F. pBQ (1µM, 1.5 min) still sensitizes currents on an rTRPV1 mutant lacking the cysteine C621G.

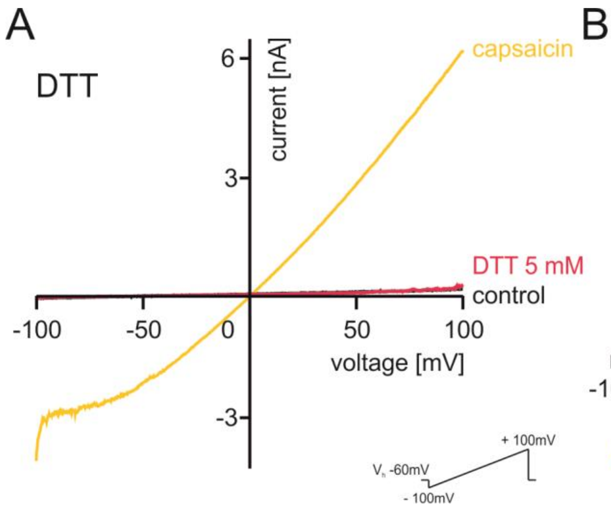

Supplemental Figure 7. Dithiothreitol 5 mM (DTT, **A**) and N-acetylcysteine (NAC, **B**) do not sensitize voltage ramp-induced currents in hTRPV1, even if applied for several minutes.

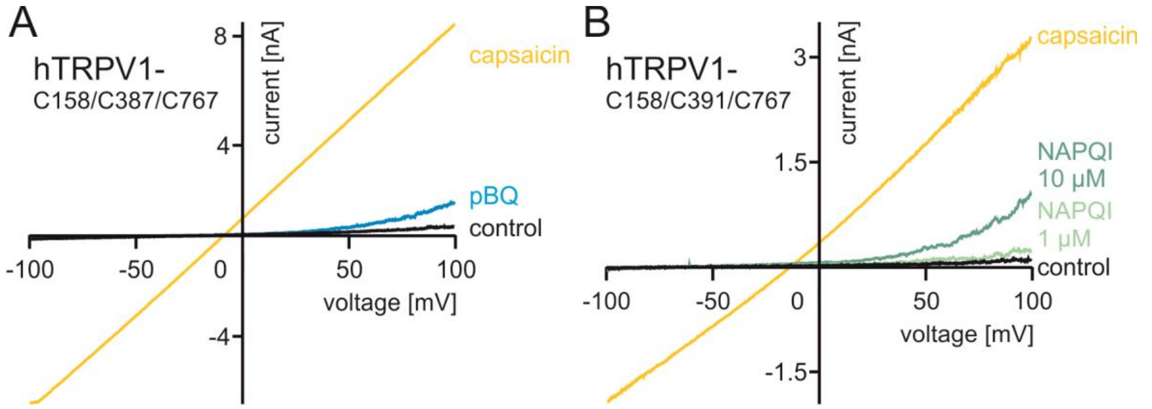

Supplemental Figure 8. A. Mutation of internal cysteines in hTRPV1 reduces sensitization of voltage ramp-induced current by pBQ (C158S/C387S/C767S-hTRPV1). B. However, higher concentrations of NAPQI (10 µM) sensitize C158S/C391S/C767S-hTRPV1 (pBQ and both concentrations of NAPQI were applied for five minutes). These cysteines are also involved in sensitization of capsaicin- and proton-induced inward currents by reactive acetaminophen metabolites.

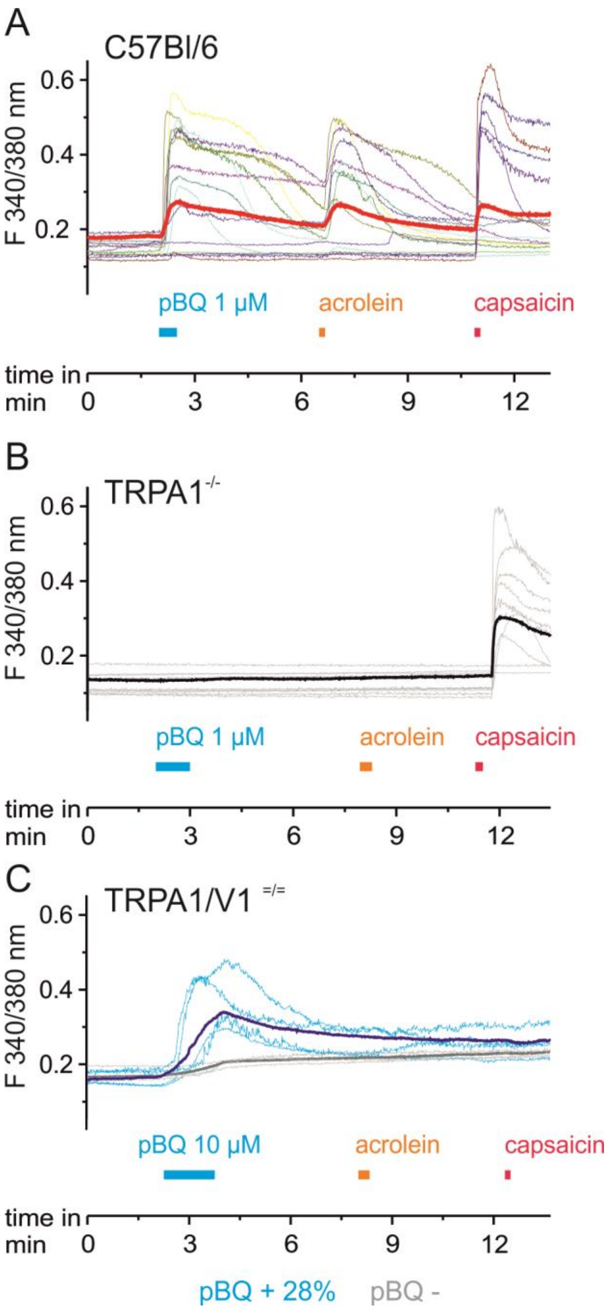

Supplemental Figure 9. pBQ induces rise in intracellular calcium in DRG neuron of TRPV1/TRPA1 double knockout mice. A. pBQ 1 µM induces calcium influx in DRG neurons of C57BL/6 mice given as mean of all measured cells (bold red trace, n = 39) and representative measurements (thin traces) due to activation of TRPA1.[5] **B.** These responses are absent in DRG neurons from TRPA1-knockout mice (n = 78). **C.** However, 10 µM pBQ also induce increases in intracellular calcium in 28 % of neurons of TRPA1/TRPV1 double-knockout mice. Traces show pBQ-responsive (blue) and neurons not responding to pBQ (gray), a small increase in the signal is observed in all cells during exposure to 10 µM pBQ (n = 303, mean bold trace, thin traces representative measurements).

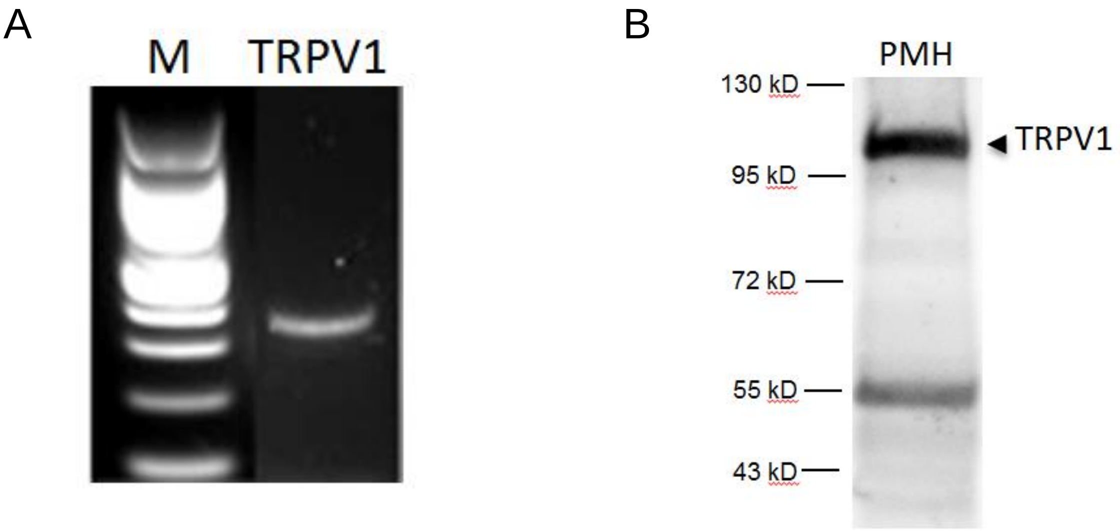

Supplemental Figure 10. Expression of TRPV1 has been shown by mRNA and western blot in HEPG2, HUH7 and primary mouse hepatocytes (PMH).[21] In accordance to these findings, we observed expression of TRPV1 by rtPCR (**A**) and western blot (**B**) in cultured primary mouse hepatocytes.

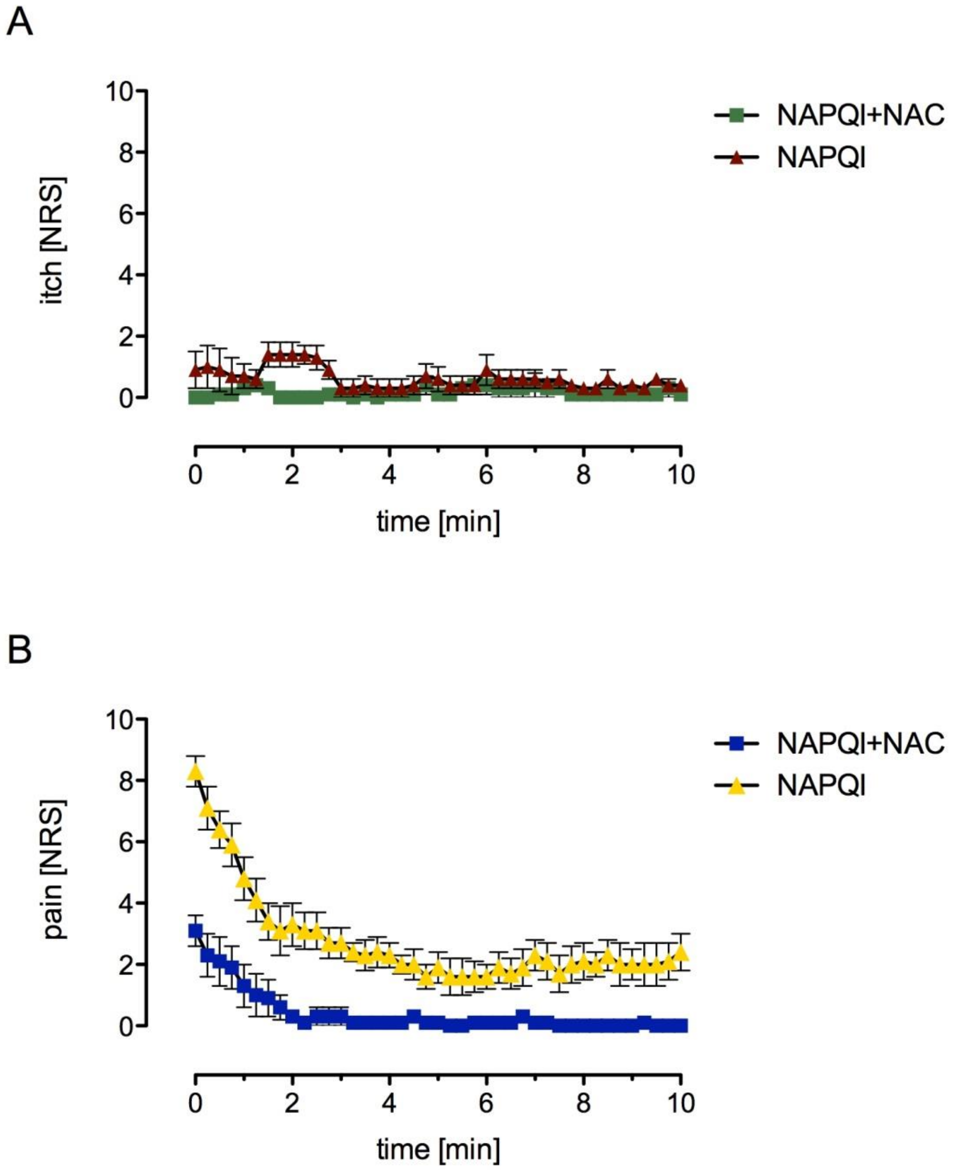

Supplemental Figure 11. Besides pain intracutaneous injection of NAPQI induces mild itching sensations. A. Magnitude and time course of NAPQI (10 mM)-evoked itch in human volunteers (n = 7) after intracutaneous injection to the volar forearm (lower panel). Itch was rated on a numerical rating scale (NRS) from 0 to 10 (a sensation of NRS 3 implies the beginning urge to scratch, mean ± SEM). **B.** Magnitude and time course of NAPQI-induced pain are given for comparison (see also figure 9A).

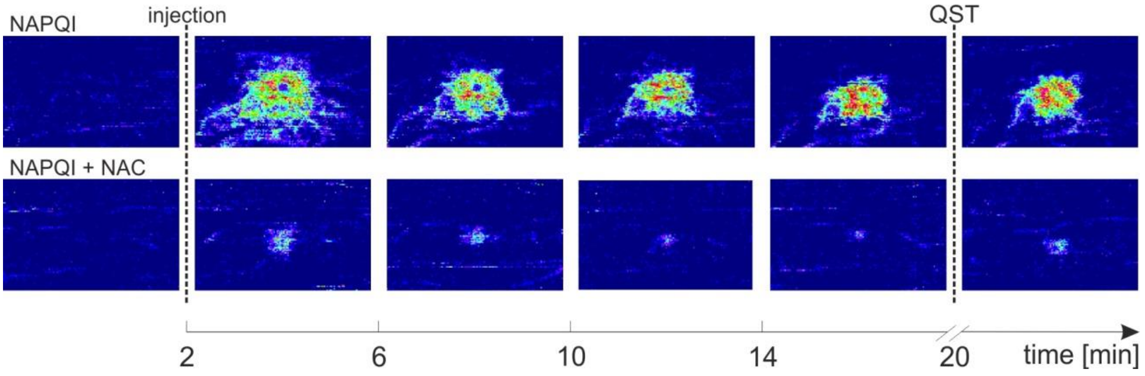

Supplemental Figure 12. NAPQI-induced axon reflex erythema. Complete series of laser Doppler scans taken before and every 2 minutes after double-blind intracutaneous injection of NAPQI or NAPQI co-injected with NAC to the volar forearm of one human volunteer. While there is a strong axon reflex erythema induced by neuropeptide release following antidromic activation of wide branching C-fibers after NAPQI injection, this response is reduced by co-application of NAC.
